# Unveiling teachers’ beliefs on visual cognition and learning styles of deaf and hard of hearing students: A Portuguese-Swedish study

**DOI:** 10.1371/journal.pone.0263216

**Published:** 2022-02-15

**Authors:** Filipa M. Rodrigues, Joana R. Rato, Ana Mineiro, Ingela Holmström

**Affiliations:** 1 Institute of Health Sciences, Universidade Católica Portuguesa, Lisbon, Portugal; 2 School of Education and Social Sciences, Polytechnic Institute of Leiria, Leiria, Portugal; 3 Center for Interdisciplinary Research in Health, Universidade Católica Portuguesa, Lisbon, Portugal; 4 Department of Linguistics, Stockholm University, Stockholm, Sweden; PLOS: Public Library of Science, UNITED KINGDOM

## Abstract

Vision is considered a privileged sensory channel for deaf and hard of hearing (DHH) students to learn, and, naturally, they recognize themselves as visual learners. This assumption also seems widespread among schoolteachers, which led us to analyse the intersection between teachers’ beliefs on deaf and hard of hearing students’ academic achievement, visual skills, attentional difficulties, and the perceived importance of image display in class. An online survey was designed to analyse the beliefs of the schoolteachers about the deaf and hard of hearing students learning in educational settings from Portugal and Sweden. Participated 133 teachers, 70 Portuguese and 63 Swedish, from the preschool to the end of mandatory education (ages 3–18) with several years of experience. The content analysis and the computed SPSS statistical significance tests reveal that surveyed teachers believe that deaf and hard of hearing students have better visual skills when compared with their hearing peers yet show divergent beliefs about visual attentional processes. Within the teachers’ perceptions on learning barriers to DHH students, the distractibility and cognitive effort factors were highlighted, among communicational difficulties in class. Conclusions about the prevalence of learning misconceptions in teachers from both countries analysed, corroborate previous studies on neuromyths in education, and bring novelty to Deaf Education field. The work of translation of scientific knowledge, teacher training updating, and partnership between researchers and educators are also urgently needed in special education.

## Introduction

The biggest concern in the Education field since the end of the 20th century is the scant recommendation of school practices based on verified facts, in contrast to the diffusion of several seductive but insufficiently informed pedagogical strategies [1]. The importance of a teacher’s experience generally is not overlooked, since can be valuable in setting priorities in each socio-cultural and educational context. However, in addition to the decision-making based on "common sense", educational practices should become more evidence-based. A bidirectional process should be established between scientific knowledge and experience, to measure, and expand the professional capacity. In the Deaf Education field, this is no exception, and the lack of research is recognized. The theoretical framework of this study is based at the intersection of the Education, Deaf Studies, and Educational Neuroscience domains. Studies have pointed that assuming that DHH students are visual learners is not helpful in the educational practical domain, leading teachers to believe in the effectiveness of visual methods and materials [2]. The DHH students often perform no better, and, to date, there is no evidence that they are more visual learners than hearing students [3]. Students’ self-perception of visual learners can reinforce teachers’ views about preferred learning styles since this approach sets the stage for the assumption that information obtained through one sensory modality is processed in the brain to be learned independently of the information received through another sensory modality. When teachers seek to invest in what they believe to be the right learning style for the student, adapting their pedagogical practice, that does not mean an improvement in students’ learning outcomes [4,5], and nothing tells us that is not the same in cases where, supposedly, have one less learning style. Concerning DHH students, instead of using unimodal and less varied semiotic resources, findings highlight the need for teachers to provide a richer context for instruction than they normally would for hearing students. Among other factors, the inconsistent educational policies, the scarce crossover of knowledge between the different scientific fields, and the insufficient evidence-based practice contribute to academic outcomes for DHH students to remain low [3]. Investigations carried out in Portugal state that DHH student education in regular schools has been occurring inappropriately, causing the isolation of these students [6]. In Swedish collaborative research, that aimed to compare the DHH pupil´s achievement in Sweden and Scotland, results show that, although social reforms and technological advances had taken place in both countries, DHH students still lag behind their hearing peers [7]. Mental and physical health already has been a subject of study, with parents and teachers reporting an increased risk of DHH children developing fatigue and stress symptoms. At different ages, fatigue can potentially compromise one’s ability to learn and result in impaired academic performance [8].

The current study proposes unveiling pedagogical beliefs that might act as an obstacle to the teachers’ practice with DHH students. To achieve a more comprehensive view of conceptions about the learning abilities and difficulties of DHH students, by investigating Portuguese and Swedish teachers’ perceptions, we addressed the following exploratory research questions:

1. Does Portuguese and Swedish teacher share the same perceptions about the language and mathematics achievements of DHH students?
2. Do most teachers believe in a visual advantage of DHH students and identify a key age at which it manifests?
3. How aware are the teachers about distractibility and cognitive fatigue factors in DHH students? Do teachers report the unequal levels of effort in attentional processes?
4. Do teachers, in both countries, identify brain-learning-related myths successfully? Are their learning conceptions about DHH students or interests in reading scientific literature protective factors from erroneous beliefs?
5. How confident are teachers about their pedagogical practices towards DHH students, and which influencing factors stand out in both educational settings analysed?

## Portugal and Sweden: What common ground in Deaf Education?

Teachers in regular schools with an inclusive orientation (mainstream schools) often feel unprepared and are largely unfamiliar with the differences of DHH students relative to their hearing age-mates, especially when considering a high heterogeneity of DHH students [3]. In the Portuguese context, there are historical reasons with implications in professional preparation for teaching DHH students and consequent levels of expertise lower than desired. Some of the gaps in Deaf Education in Portugal are due to a slower and fragmented implementation of more effective teaching methods, pointing to the lack of updated practical knowledge produced in Europe [9]. A close relation between Portugal and Sweden started when Pär Aron Borg, founder of the first Deaf Institute in Sweden, was invited by to lead the Instituto da Luz for Deaf Education in Lisbon, in 1823. When Borg later returned to Sweden, his brother Johan Borg, took over the direction of the Institute until the date of his death, in 1833. The used pedagogy continued following the use of sign language, but new setbacks of political order would lead to the Institute extinction Portugal and Sweden have shared important historical aspects in Deaf Education since the 19th century, and sign alphabets still have similarities nowadays [9]. Sweden has a long time been regarded as in the frontline of Deaf Education and the Swedish sign bilingual education has been a role model for other sign bilingual attempts worldwide, with Portugal included. Sweden was the first country in the world to give a sign language the status of a language [10].

Therefore, these countries have a common rooted system, but which had undergone decisive conceptual changes, namely after the II International Congress on Education for the Deaf in Milan, in 1880. At this congress, it was decided to exclude Sign Language from Deaf Education in seven of the nine present countries (among them was Portugal), having chosen for the oral/ speech teaching method. Except for Sweden and the United States of America, this method prevailed until the XXI International Congress in Vancouver [9]. Between these two international events, separated by 130 years, the repercussions felt in the several countries that shaped their educational systems. It is based on this deaf education past, that is relevant to verify points of convergence and divergence in the Portuguese and Swedish teacher’s perceptions about deaf students learning.

Despite the Portuguese disadvantage with a gap of more than ten years between the recognition of the Portuguese Sign Language and a delay in the implementation of an inclusive bilingual policy, it is pertinent to take a picture of the deaf education beliefs in both countries.

## Attending visual access under misbeliefs in learning styles: What barriers for DHH students?

Visual cognition involves high-level cognition dimensions linked to the perception, memorization, and analysis or interpretation of phenomena and objects, enhancing relations between seeing and thinking, and thus, learning. Research shows that visual cognition processes in DHH students are carried out progressively and intentionally and, for it to occur it is necessary to resort to oneself curiosity and critical thinking skills so that meaningful learning is built [11]. Visual cognition is not acquired only through the sense of sight or the belief in an automatism without cognitive intentionality.

There are potential problems, that might result from teaching strategies, that could be reducing the multidimensional options of learning processes by assuming one preferred sensory modality [12–15]. Available data clarifies that students can show preferences about their learning styles, but these have no relation to their learning outcomes [14]. Students do not process information more effectively when they received the information accordingly to a specific learning style [4,5,15,16]. Some dimensions that should be considered when related to the investment in unimodal teaching strategies relate to poorer environments for learning. Nevertheless, even if the effectiveness of multimodal (and multilingual) communicative impact is recognized, there are still challenges in the school context to consider for DHH students. The role of visual attention and in DHH students has been considerably studied, but less is known about the effects of the difficulties in maintaining attention in the classroom context when trying to reconcile different sources of information [17]. Previous studies have shown some differences between DHH and hearing individuals, not in terms of visual acuity, but the dimension of attention to what is perceived in the surrounding environment, such as peripheral visual attention. Furthermore, research has shown that deaf signers have a heightened ability to detect the direction of movement in the periphery of vision, for example, are faster in shifting visual attention compared to hearing individuals [18–20]. Focusing on the issue of distractibility in DHH students, research establishes comparisons between the auditory and visual distractions, stating that DHH students pay attention to phenomena that visually distract them, just as hearing students are sensitive to potential auditory distractions [19]. Also, it is reported that peripheral visual attention abilities that are developed and learned over time are not found in children before eleven to thirteen years of age, coinciding with a growing awareness of selective attention mechanisms. Only at around fourteen to seventeen years old, do DHH youngsters detect and differentiate between static and mobile stimuli in the periphery and shape their behaviour facing distracting effects on the surrounding environment [21–23]. DHH students are considered to struggle in school tasks as being in a constant situation of splitting attention (due to a single channel for visual input of classroom information in contrast to hearing students who use dual channels—auditory and visual). The challenge increases if teachers and sign interpreters do not consider the adverse effects of high reaction/attention span on DHH students’ visual field, which implies difficulties in maintaining attention and cognitive load in the case of simultaneously scattered stimuli [24]. Pragmatic examples reveal episodes when DHH students lose access to the information transmitted in class due to the difficulty of visual access [21,25]. Concerning sources of distraction in the context of Deaf Education, there is available bibliography about effective multimodal teaching methodology, i.e., on teachers’ and the sign language interpreters’ combined performances and some aspects of the classroom environment. In most cases, creating a suitable physical environment for deaf students will involve designing a space without excessive visual noise to promote the students’ focus on the proposed tasks [21]. Compared to hearing students, DHH students have fewer opportunities to take breaks. While the hearing students will pause spontaneously when listening to the teacher, such as drawing, or looking at the images of the book, whereas DHH students do not feel the same ease in abstracting from what the teacher or interpreter transmits, as both constantly deliver visual information. Potential fatigue and distractibility implications from high-loaded visual environments should be more discussed within investigations in Deaf Education similar to those which have already been done in educational contexts of hearing students [25]. As already seen in a recent experimental study, hearing children are constantly exposed to visually enriched environments such as paintings, posters, or other objects that appear exposed on the walls, since the early years of schooling. Researchers concluded that a high-loaded visual environment could affect children’s cognitive performance and different levels of exposure to visual elements may contribute to the distraction of children, resulting in impaired learning outcomes. Other research on fatigue levels, also reveals the effect of hearing loss on subjective reports of fatigue in school-age children [26]. Therefore, without criteria specifically designed for the education of DHH children, accumulated tiredness and fatigue need to be studied to better understand the academic performance particularities of DHH students.

## Neuromyths are rampant in schools

Based on the literature that discusses the neuroscientific misunderstanding in education, teachers seem not immune from neuromyths about how learning proceeds. The oversimplification of brain research or the inappropriate transferring for classroom practice is some of the problems pointed out [27,28]. Previous research put into analysis the misconception of preferred learning styles in different countries [29] and the widespread idea that children should be taught according to one of the three main learning profiles (Visual-Auditory-Kinesthetic, VAK/learning styles) is one of the most persistent myths in schools’ contexts [4]. The erroneous application of neuroscientific research findings in educational practice has been highly discussed and more evidence-based practices are needed to accurately transfer brain research findings to classroom practices [30]. The origin of neuromyths comes in part from the enormous extrapolation of scientific data, for example, it is known that visual, verbal/auditory, and kinaesthetic information is processed in different parts of the brain, but these brain structures are highly interconnected and cross-modal transferring of sensory information exists. It is, therefore, incorrect to act on the assumption that each student only uses one sensory modality to learn [31] and nothing indicates that this occurs differently in cases with sensory channels restrictions, although the literature is scarce on the subject.

Recent studies [15,32] showed that worldwide teachers fail to distinguish myths from facts and, besides their interest in brain research knowledge, the scientific findings often have been misinterpreted by educational professionals being away from evidence-based practices. Despite the wide acceptance of the importance of neuroscience approaches in education, no studies on neuromyths involving teachers of DHH students have been performed.

## Materials and methods

### Participants

A total of 133 schoolteachers (70 Portuguese and 63 Swedish), with expertise working with DHH students, participated in this study (Table 1). Regarding the educational context, 84.3% of the Portuguese teachers work at public mainstream schools with bilingual education for DHH students (reference school for bilingual teaching of deaf students–or EREBAS). Approximately half of the Swedish teachers (49.2%) work at sign bilingual deaf schools and the other half work with DHH students in mainstream schools. In both countries, practitioners report teaching or supporting (Special Education Needs) in the context of classes for deaf students or in integrated deaf classes (mixed classes with hearing students and DHH students).

**Table 1:** Demographic data of Portuguese and Swedish teachers (N = 133).

	Portugal (n = 70) %	Sweden (n = 63) %
**Age**		
23–35 years old	7.1	15.9
36–45 years old	27.1	39.7
46–55 years old	31.4	25.4
56–65 years old	34.3	19.0
**Teaching Position**		
Special Education teacher (SEN)	35.7	20.6
Sign Language teacher	12.9	6.3
Pre-school teacher	2.9	0.0
Primary teacher (5-9y)	7.1	12.7
Teacher (10-12y)	5.7	33.3
Teacher (13-15y)	10.0	23.8
Teacher (16-20y)	25.7	3.2
**Learned Sign Language**		
No answer	21.4	11.1
0–3 years old	14.3	23.8
4–7 years old	1.4	4.8
8–11 years old	4.3	4.8
12–15 years old	5.7	3.2
16–19 years old	1.4	9.5
>20 years old	51.4	42.9
**Teaching Experience with DHH students**		
No answer	1.4	0.0
< 5 years	31.4	20.6
Between 5–10 years	12.9	15.9
Between 10–20 years	35.7	27.0
> 20 years	18.6	36.5

Concerning the question “Learned Sign Language at what age?”, some teachers did not give us an answer. Despite this, the percentage of teachers who learned sign language aged 20 or over is relevant to this study. The Portuguese sample has the highest percentage of older teachers, observing a significant difference between the two countries with 65.7% of Portuguese teachers between 46 and 65 years old, while the Swedish sample is 44.4% (U = 1643.50, p = 0.008).

### The online survey

We designed a questionnaire for this study and conducted an online survey in Portugal and Sweden to obtain an exhaustive picture of the teacher’s perceptions concerning the process of learning of DHH students linked with their putative beliefs in learning neuromyths. Teachers from both countries completed the same questionnaire, but in their native language. Our survey covers a wide range of cross-cultural issues, but only the items related to the research questions previously addressed will be described in this study. The items were organized into three main parts. In the *First Section*, we collected personal data about the teachers, such as, the age, area of the country, educational qualifications, school position, context, and level of education and teaching time with DHH students. In this section, it was also asked if respondents usually consulted scientific literature in the area of neuroscience, education, and deafness, as well as a question about the level at which respondents placed DHH students in the Portuguese/Swedish language and Mathematics subjects compared to the hearing peers.

The *Second Section* aimed, through open and closed questions, to assess the teachers’ conceptions and practices about DHH students’ visual perception, visual attention, and visual memory skills, as well the impact of visual strategies in the classroom. In this section, the closed response options were given using a 5-point Likert scale from "Strongly Disagree" to "Strongly Agree", and for open-ended questions, a text box was available to write on.

In the *Third Section*, dedicated to beliefs about learning, the following five statements were presented:

1. “Students learn best when they receive information in their preferred learning style”
2. “Students show preferences in the way they receive information”
3. “Environments that are rich in stimuli further develop the brain”
4. “There are critical periods for learning”
5. “Children must acquire their mother tongue before a second language”,

Our statement selection process resulted in a panel of experts (five faculty members with expertise in cognitive neurosciences, educational psychology, science education, and linguistics), who analysed several statements based on rating: a) clearly wrong; b) mostly wrong; c) no clear decision; d) mostly true and e) clearly true. Here, our focus was on the learning styles items, but to balance it, other learning-related topics we added. Five statements, based on neuroscientific facts and fiction, were selected considering the framework of the present study. Statement 1 is not supported by the literature since there is no scientific proof, but for statement 2 previous research is accepted. The claim of statement 3 has found controversial findings in the literature and the thesis from statement 4 was considered true in the past, but ultimately refuted based on brain neuroplasticity. Finally, statement 5 is highly not substantiated by the research findings. In this section, open-ended questions were also added to inquire teachers about the confidence in their pedagogical decisions with their DHH students, and the need for some lifelong learning modalities.

### Procedure

The main questionnaire has an estimated fifteen-minute completion time, but the time dedicated to answering optional open-ended questions is dependent on each participant. The questionnaire was revised in both countries by four collaborating teachers, and a final version was sent to the principals of the mainstream public schools (in the Portuguese case) and the National Agency for Special Needs Education and Schools (in Sweden). The questionnaire (S1 and S2 Files) was widely disseminated on schools and social networks by the institutions involved in this research project, and data collection took place between 2019 and 2020. Electronic informed consent was applied under the approves of the Ethics Committee for Health of the Catholic University of Portugal (Ref. Number 18) The answers were given freely, no dropouts to report, and data processing was carried out following the required conditions of confidentiality.

### Data analysis

The representative number of teachers who teach DHH students is still unknown, so we are unable to ascertain how many may have received the questionnaire link and not responded. The self-select samples from both countries included participants who taught exclusively DHH students (sign bilingual deaf schools in Sweden) and others who worked with both DHH and hearing students (mainstream schools from Portugal and Sweden).

Statistical Package for Social Sciences (SPSS version 25) software was used to analyse the collected quantitative data. The Mann-Whitney test was used whenever the two groups were compared in variables with a qualitative scale. When comparing with nominal qualitative scales, the chi-square test was used whenever the requirement for its use at the level of expected frequencies was met (< = 20% of expected frequencies <5). Fisher was used as an alternative to chi-square in situations where there were more than 20% with expected frequencies below 5. The Web QDA software was used as a technical-methodological procedure for qualitative analysis of the open-ended questions, where the category, content, and semantical analysis of the responses were attended.

## Results

### Teachers’ perceptions (on DHH students’ academic achievement, visual skills, attentional difficulties)

**Fig 1:**
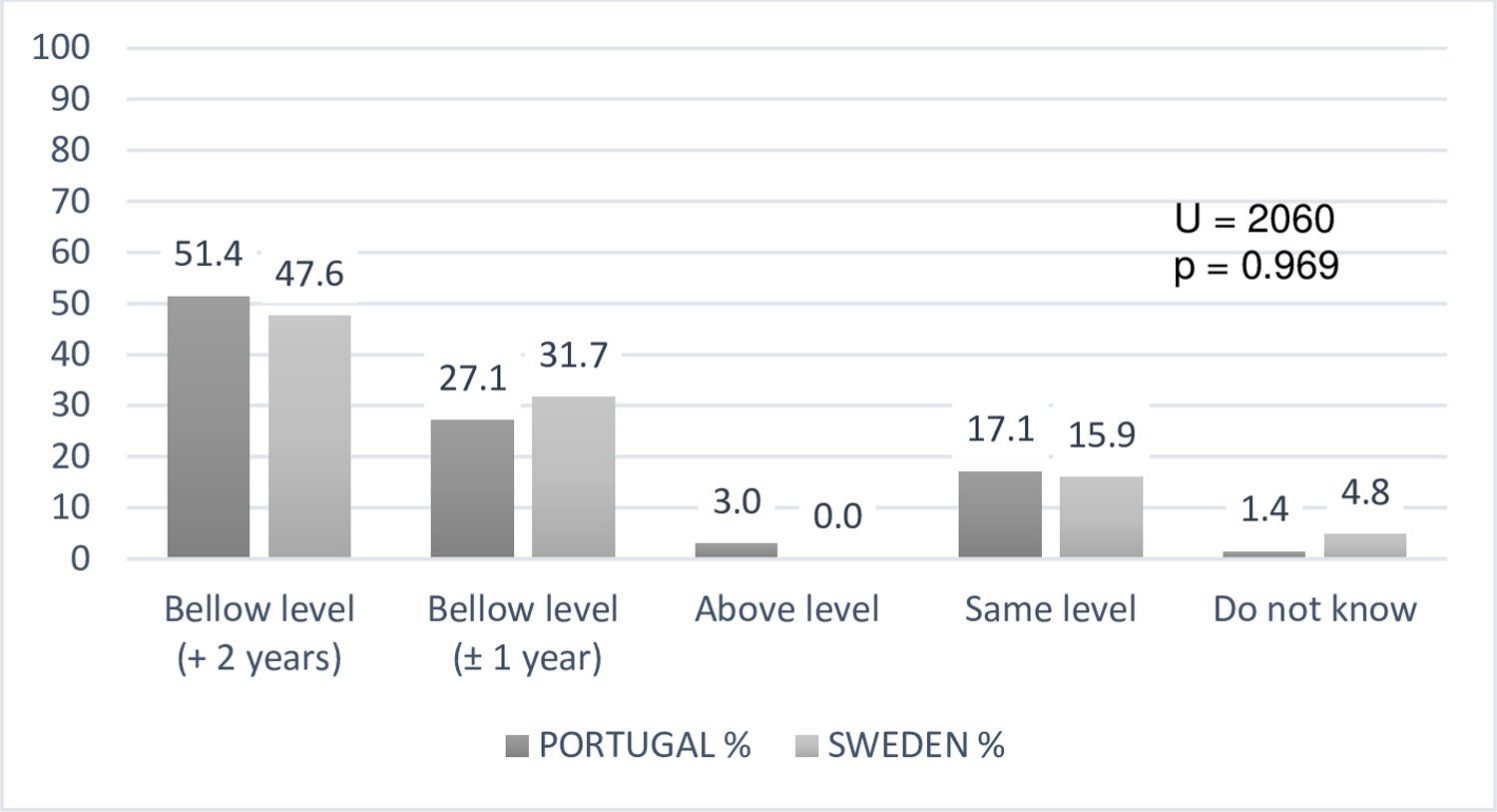
Teachers’ perceptions about the language performance levels of DHH students (N = 133).

About our first research question on teacher’s conceptions about the learning achievement of DHH students, a comparative overview of collected data places Portuguese and Swedish students in lower levels of language achievement with relevant values for the “below level for more than 2 years”, when compared with other options (Fig 1).

**Fig 2:**
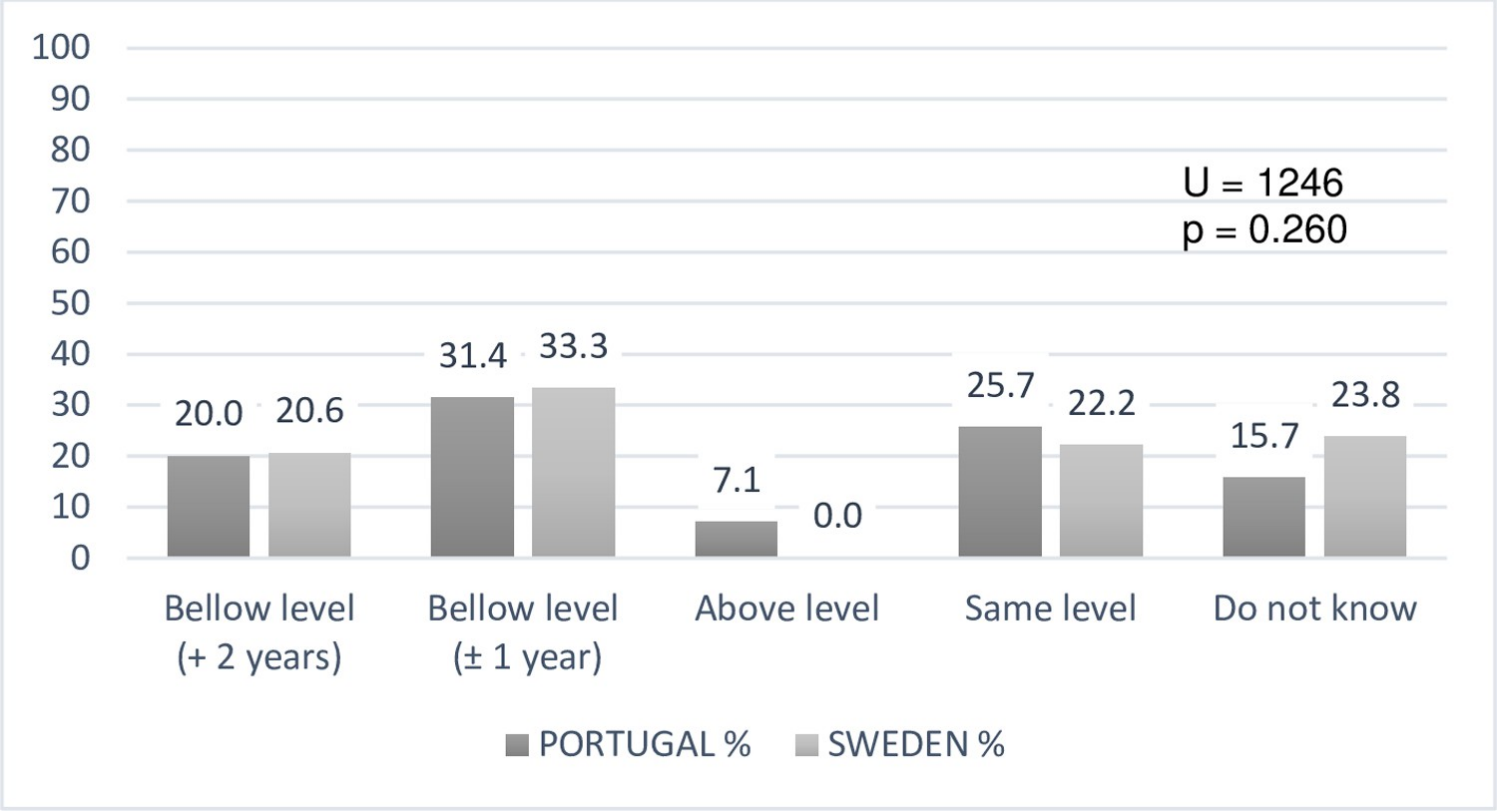
Teachers’ perceptions about the mathematics performance levels of DHH students (N = 133).

This gap about following the curriculum is not so marked concerning mathematics, yet teachers recognize a delay of at least 1 year in learning. In comparative terms, by countries, there are no relevant statistical differences in the math domain (Fig 2).

**Fig 3:**
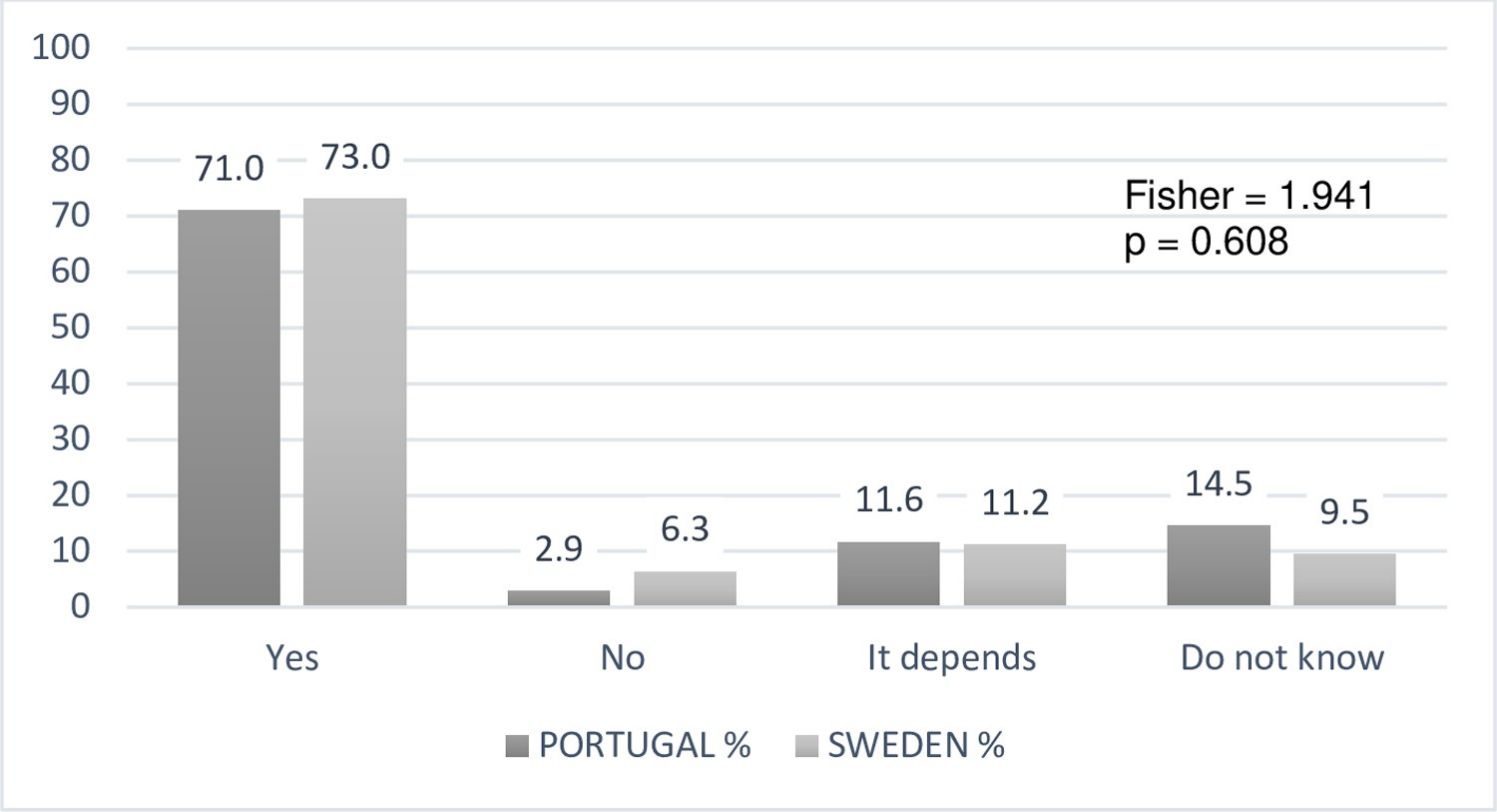
Teachers´ perceptions about DHH students’ visual skills (N = 133).

Regarding the second research question that focuses on teachers’ perceptions of enhanced visual skills for DHH students, both samples acknowledge this advantage in DHH students but showing no relevant statistical differences between countries (Fig 3). Here, the question presented was: “According to your opinion, is it likely that DHH students have better visual skills (e.g., visual perception, visual attention, and visual memory), compared to their hearing peers in the same age group?”.

**Fig 4:**
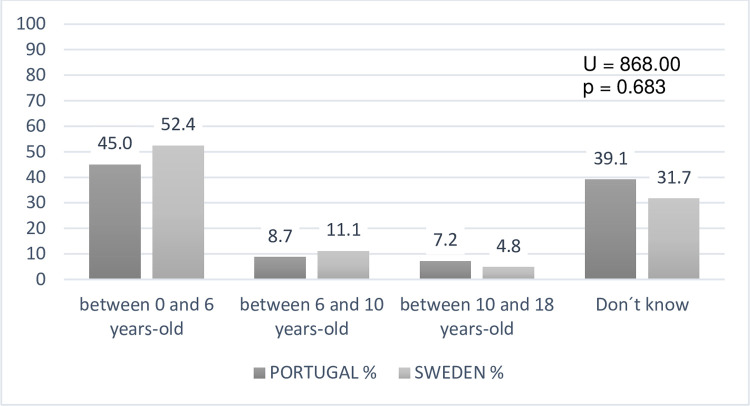
Teachers’ perceptions about increased visual skills manifestation ages (N = 133).

In the case of teachers’ agreement with an advantage in DHH students’ visual skills, data, in terms of the age rate, indicates there is a similarity between the teacher’s answers from both countries. Highest in the 0–6-year-old age group (U = 868.00; p = 0.683), and lowest in the 10 -18-year-old age group (Fig 4). Also noteworthy is the "Do not know" answer, which is around 30% in both countries.

**Fig 5:**
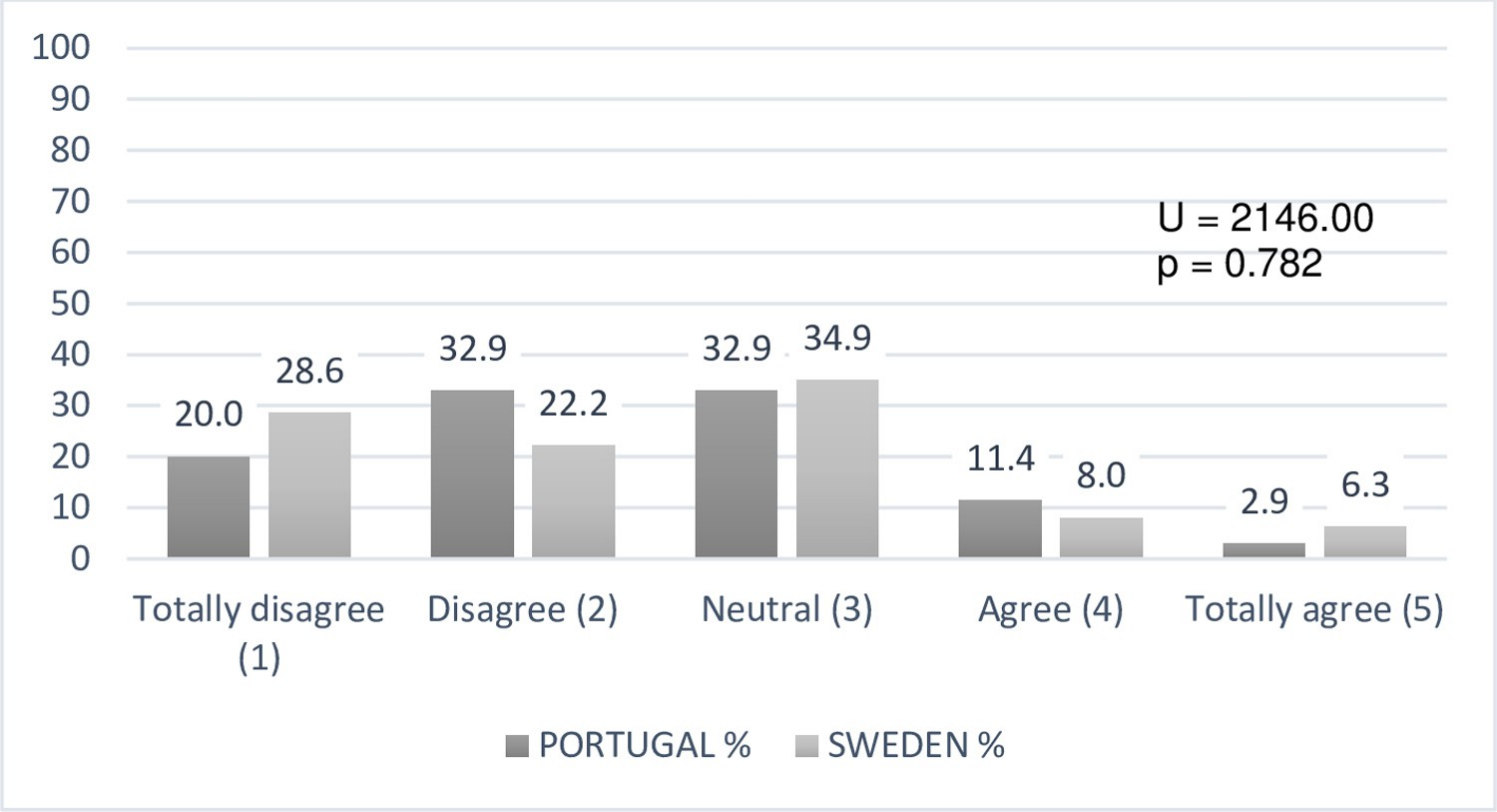
Teachers’ conceptions about DHH learners’ attentional difficulties in class (N = 133).

Concerning the teachers’ perceptions about the difficulties of DHH learners, the third research question established the conceptual bridge between how aware teachers are about distractibility and cognitive fatigue factors in DHH students. We tried to gauge teachers’ perceptions of the possible difficulties that DHH students may demonstrate compared to their peers in the classroom, as well as the awareness of DHH students’ problematic fatigue issues by asking teachers: “In your opinion, deaf children/pupils have more difficulty maintaining visual attention than their hearing peers? And why?” (Fig 5).

Comparatively, the results showed no statistically significant differences in both samples. Using the content analysis method, it was possible to encode the answers through the identification of categories from text units of all received responses to “And Why?”. Here, the participants’ answers in both countries (Portugal: n = 39 and Sweden: n = 38) were coded in two comprehensive categories: 1) “Fewer difficulties in maintaining attention” and 2) “More difficulties in maintaining attention”.

For the first coded category, we identified text references that suggested five subcategories: a) interest and motivation; b) habituation/training; c) focus on images; d) mastery of the sign language, and e) visual acuity. From these subcategories, the a), b) and c) accounted for the largest number of text units, with fewer difficulties in maintaining attention printed to factors related to the interest and motivation of the students. However, this was a reason only presented by the Portuguese sample, with no references to it by Swedish teachers. The “habituation/training” factor was mentioned in both countries (Portugal: n = 3; Sweden: n = 3), as the justification that the DHH students “focus more easily on images” (Portugal: n = 3; Sweden: n = 2).

In the second category (more difficulties), the factors a) distractibility; b) tiredness, and c) teaching performance were the three subcategories prevalent in both countries. The reference to “distraction” (Portugal: n = 8; Sweden: n = 3) was the highest weighted impact pointed to the difficulties of DHH students. Issues related to “tiredness” were significantly mentioned by Swedish teachers (Portugal: n = 3; Sweden: n = 6).

**Fig 6:**
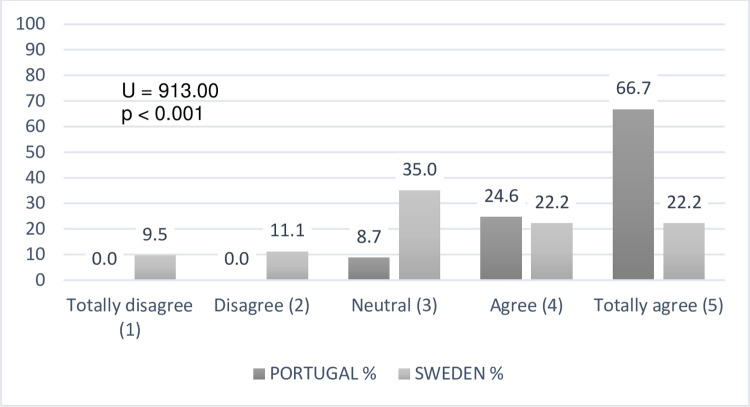
Teachers’ conceptions about the importance given to the visual material exhibition in classroom walls (N = 133).

Concerning the teachers’ degree of agreement with the statement "The exhibition of a diverse set of images on the classroom walls is important to reinforce DHH students learning", data showed statistically relevant differences (Fig 6), with the Portuguese sample agreeing more markedly (U = 913.00; p <0.001).

### Teachers´ beliefs (demographic data)

Concerning our fourth research question, we presented a set of 5 statements based on neuroscientific fact and fiction (Table 2), which two statements (1 and 5) are consensually recognized as a neuromyth, the other two statements (3 and 4) are under scientific controversial rating but also claimed as a neuromyth, and one statement (2) is considered as a fact. Participants in both countries were asked to classify each statement as a "myth", a "fact" or choosing the "I do not know" option. Statistical data reveals that there is no significant difference between the two countries in total false beliefs [t (131) = 1.090, p = 0.278], although in the teaching experience variable a joint analysis was performed to realize the effect on all teachers’ beliefs.

**Table 2:** Portuguese and Swedish teachers’ answers concerning myth/fact statements (N = 133).

*Statements*	*Incorrect %*		*Correct %*		*Don´t know %*		*Statistical* *test*	*P value* ^a^
	PT	SW	PT	SW	PT	SW		
1. Students learn best when they receive information in their preferred learning style (M)	81.4	90.3	7.2	1.6	11.4	8.1	Fisher	2.443
2. Students show preferences in the way they receive information (F)	2.9	12.7	85.7	66.7	11.4	20.6	*χ2*	7.620
3. Environments that are rich in stimuli further develop the brain (C)	85.5	88.9	1.5	3.2	13.0	7.9	Fisher	1.749
4. There are critical periods for learning (C)	31.4	24.2	40	53.2	28.6	22.6	*χ2*	2.086
5. Children must acquire their mother tongue before a second language (M)	35.7	31.7	34.3	57.1	30.0	11.1	*χ2*	9.614

^a^ *p* values are NS (or p> 0.05). (M) Myth; (F) Fact; (C) Controversial Myth.

In a preliminary analysis, performed separately by sample group, the professional experience does not reveal to have an impact on teachers’ neuromyths beliefs: in the Portuguese sample there are no differences between those who have experience of up to 10 years (M = 2.29, SD = 1.07) and more than 10 years of experience (M = 2.63, SD = 0.97) in the total of false beliefs [t (67) = -1.38, p = 0.170] and similar results were observed in the Swedish sample (M = 2.39, SD = 1.11; M = 2.82, SD = 0.98, respectively) also without significant difference to report [t (61) = -1.60, p = 0.114].

Notwithstanding the above results, we performed a combined Student´s *t*-test for the all sample and when comparing merged groups less experienced (*n* = 54, M = 2.33, SD = 1.08) with those with more than 10 years of experience (*n* = 78, M = 2.73, SD = 0.98), significant differences were found for p<0.05, with a higher rate for false beliefs for the group with more experience [t (130) = 2.200, p = 0.030, d = 0.40].

Regarding the comparison between the age at which the teachers learned sign language, the Student´s *t*-test did not reveal, in both samples, significant differences between the two groups. In those who learned sign language up to 15 years old (Swedish M = 2.86, SD = 1.12; Portuguese M = 2.47, SD = 1.23) and those who learned after completing 15 years old (Swedish M = 2.64, SD = 1.01; Portuguese M = 2.59, SD = 0.95) in the total of beliefs [t (54) = 0.749, p = 0.457; t (53) = - 0.357, p = 0.723, respectively).

### Teachers´ beliefs (visual skills and acceptance of neuromyths)

Here, exploratory data analysis also suggested interesting results through crossing items from the questionnaire, as the DHH student’s visual skills teachers’ perceptions, and the neuromyth of a preferred learning style (Table 3).

**Table 3:** Percentage of teachers’ perceptions (DHH visual belief crossed with learning styles neuromyth) (N = 133).

“Students learn best when they receive information in their preferred learning style”								
**Better** **visual** **skills?**	**PT %**			**Statist. test**	**SW %**			**Statist. Test**
	Yes	Fact	90.0	Fisher p = 0.004	Yes	Fact	89.1	Fisher p = 0.301
		Myth	6.0			Myth	0.0	
		Do not Know	4.0			Do not Know	10.9	
	No/ Do not Know	Fact	60.0		No/ Do not Know	Fact	88.2	
		Myth	10.0			Myth	5.9	
		Do not Know	30.0			Do not Know	5.9	

The Fisher’s test revealed that Portuguese teachers that agree with DHH students having better visual skills than hearing peers have also significantly false beliefs about learning styles (p<0.01). In the Swedish sample, no significant statistical differences were verified between DHH students’ better visual skills perceptions and misconceptions in learning styles (p = 0.301).

The Portuguese association between DHH *have* better visual skills *vs*. DHH *have no* better visual skills, within the total neuromyths score, shows differences between the group that responded affirmatively. The Portuguese sample have a higher average on the total of false beliefs (M = 2.68) than the group that answered negatively (M = 1.90), [t (131) = 3.229, p = 0.002]. In the Swedish sample, there are no differences between the better visual skills perceptions in accounting for more learning false beliefs in total (M = 2.65), [t (61) = -0.179, p = 0.858].

### Teachers’ beliefs (scientific literature reading)

With our fourth research question, we also wanted to verify if the identification of neuromyths was related to the scientific literature reading habits in the Deaf Education and Neuroscience related domains. Fisher’s tests did not reveal significant differences in the Swedish sample. In the Portuguese sample, those who claim reading scientific literature have significantly higher false beliefs (M = 2.67) than those who do not (M = 2.00), (t = -2.892, p = 0.007). The chi-square test revealed differences between those who search for scientific sources, especially in the bilingualism neuromyth (X^2^ = 6,117, p = 0.047).

### Teachers’ confidence degree

**Fig 7:**
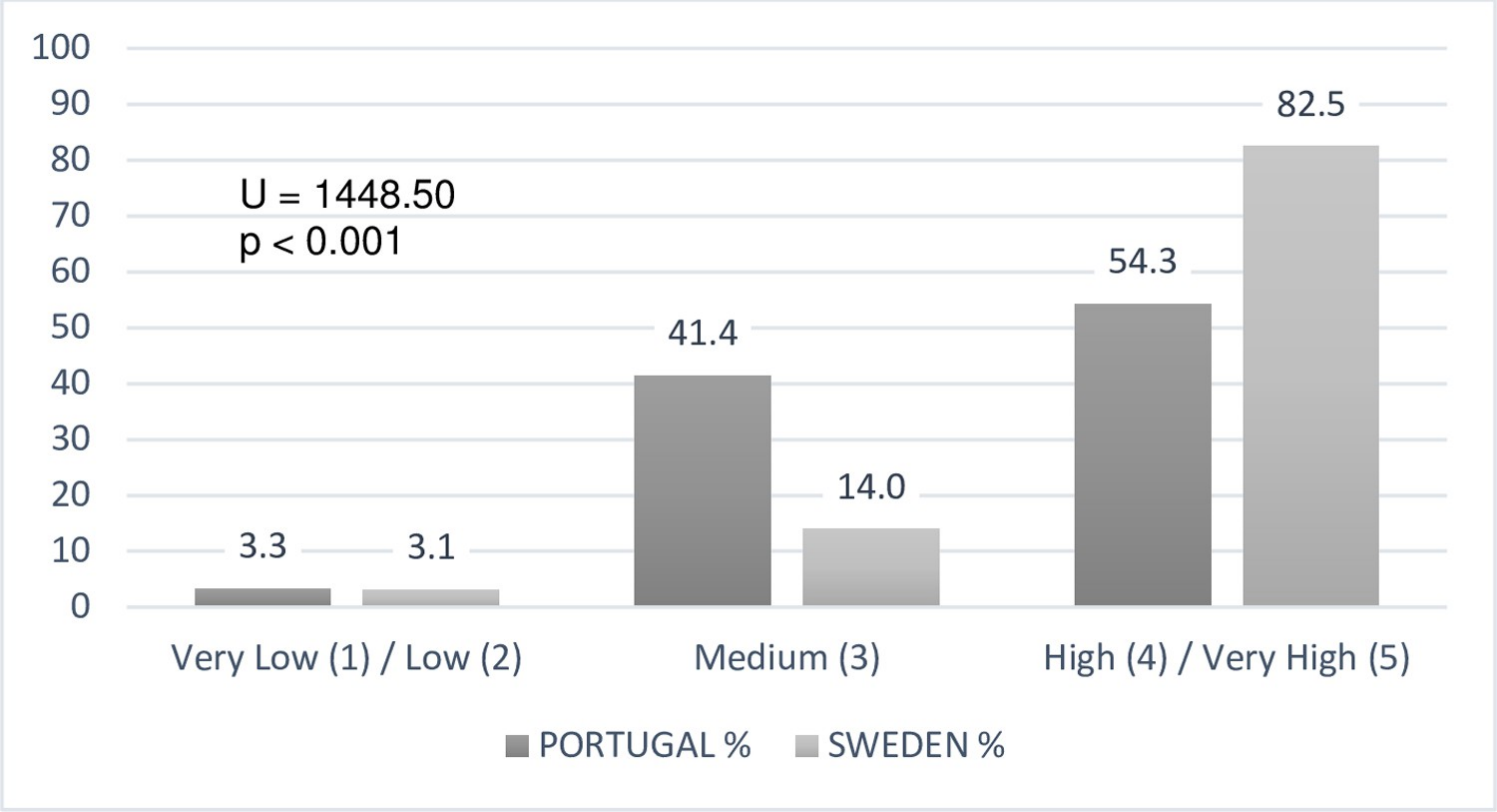
Teachers’ confidence degrees about their pedagogical choices (N = 133).

As to our fifth and last research question, the teachers’ confident degrees about their pedagogical practices towards DHH students, revealed differences between the samples analysed (Fig 7).

The Swedish teachers feel much more confident in their pedagogical options with DHH students (a higher number of extremely confident responses) compared to Portuguese teachers. Concerning the content analysis, we found that the lowest confidence levels (1 to 3) present seventeen descriptive justifications by Portuguese teachers and four by Swedish ones. Three main categories were coded: 1) ongoing teacher training needs (e.g., Sign Language training, Portugal, n = 4; Sweden, n = 1); 2) heterogeneity of students’ difficulties, as the degrees of deafness (profound deafness) and other cognitive or communication impairments (Portugal, n = 3; Sweden, n = 3), and 3) difficulties in adapting strategies that only appear manifested by Portuguese teachers (n = 10), with text units showing factors such as an excessive number of students per class (n = 3), "noise" in communication or lack of feedback due to the need for sign language interpreters in the classroom (n = 2), insufficient resources (n = 1), academic results below expectations (n = 2), DHH students´ families involvement in children’s schooling (n = 1) and the early intervention on school success (n = 1).

**Fig 8:**
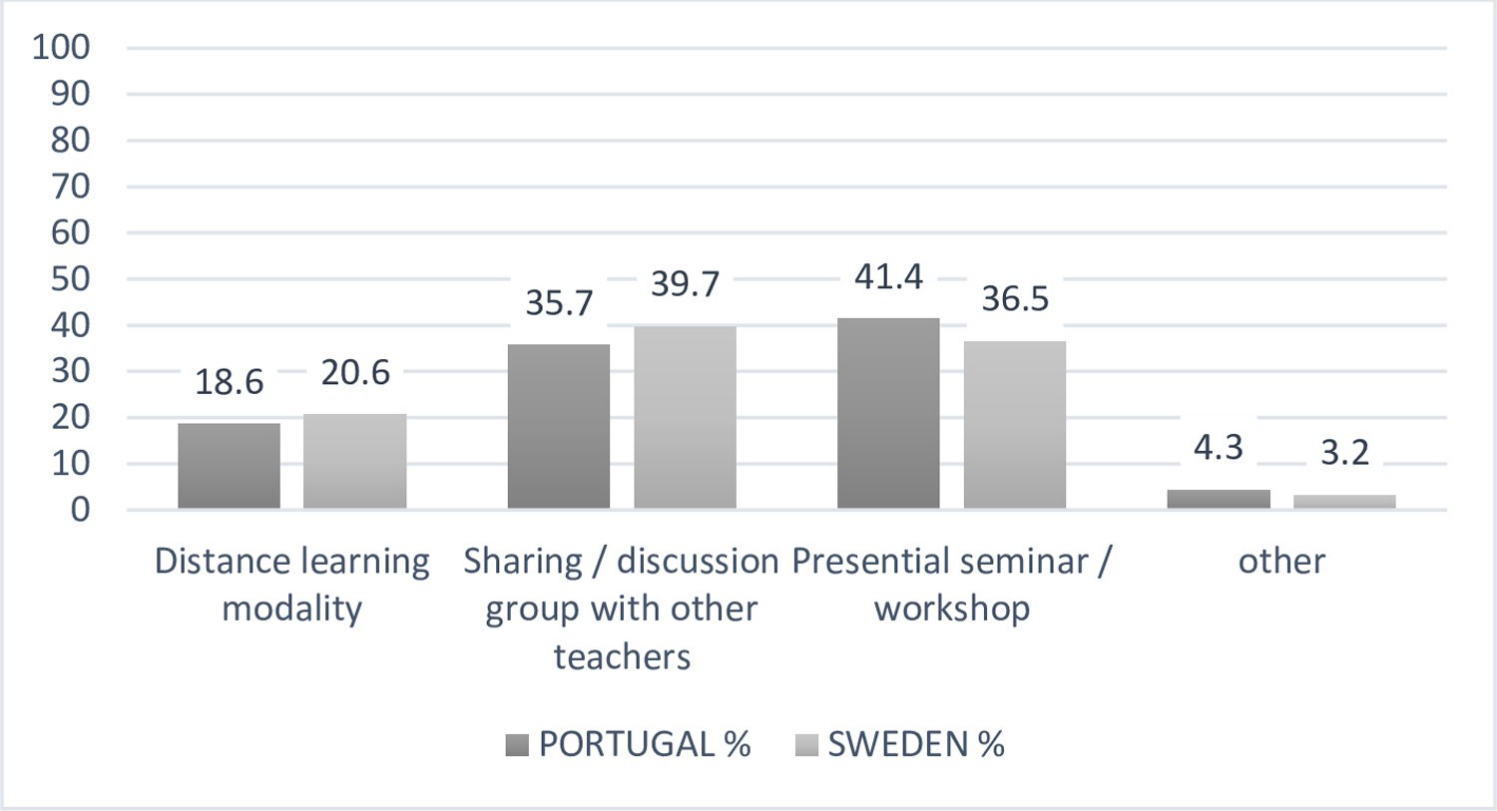
Training modalities selected by teachers (N = 133).

Our questionnaire ends with the type of training that teachers would like to attend in the future, and we found that their preferences, in both countries, are mostly divided between discussion groups and face-to-face workshops (Fig 8).

## Discussion

To date, the teachers’ views about how DHH students learn were undiscussed, and, with this study, we presented an exhaustive picture of perceptions and beliefs that might interfere with teachers’ practices. To obtain a more complete framework for analysis, we studied teachers from two countries (Portugal and Sweden) that shared a past in the teaching of DHH children and currently have their own cultural and educational particularities.

Firstly, the teachers’ perceptions in both educational settings analysed, acknowledge a gap in language and mathematics when comparing to DHH students’ hearing classmates, especially in the language acquisitions (more than 2 years). Despite the high abilities of teachers or sign language interpreters, the view that DHH students still leave the mainstream classroom with less content knowledge than their hearing peers persists, although literature conflicts on what cognitive domains factors cause the academic achievement variability of DHH students [2,33,34].

Most of the academic outcomes of DHH children’s studies have been conducted in the United States, United Kingdom, and Sweden [34]. Portugal has been on the side lines of these investigations and a comprehensive state of art about DHH student’s achievements is needed.

Relatively to the belief in a visual advantage concerning hearing peers, our results reveal an enhanced visual skill for DHH students by the teachers’ view, confirming the widespread belief in a compensatory mechanism that develops the sense of vision due to hearing deprivation and which is activated in early childhood. Although a relationship of causality between deaf enhanced peripheral visual abilities and auditory sensory deprivation (due to the auditory cortex responses to visual and somatosensory input in visual cross-modal re-organization) is documented [35], little is known about the timings in which this re-organization may occur all the variables involved and the degree to which this improvement in visual skills depends on learning. Consistent findings indicate that auditory deprivation does not necessarily lead to enhanced visual memory [36]. Considering that most of our sample of teachers believe that the DHH visual increased abilities are manifested at very early ages, contradicting scientific evidence that suggests that visual cognition mechanisms are, to some extent, the result of individual and adaptive development mechanisms "learned" in time [21], teachers might have been implementing pedagogical-didactic options that do not consider the particularities of DHH students’ development trajectory. Moreover, we can hardly find a deaf student “standard” profile as different hearing profiles, varied residual hearing, and differences due to the use of cochlear implants [37,38] are common.

The distractibility and cognitive fatigue issues were perceived by teachers as identical in both samples analysed with their acceptance that these difficulties could occur more frequently with DHH students. The content analyses revealed that Portuguese teachers focus the distractibility as the main problem, while tiredness was significantly mentioned by Swedish teachers. This suggests that teachers recognize the “visual noise” as a barrier to learning and specific difficulties related to dispersion of attention by non-relevant visual stimuli must be considered. Teachers’ perceptions on tiredness and greater effort of DHH students coincided with one of the most recent and relevant international debates. Although the prior investigation suggests that when the materials exposed in the classroom increase, also the attention difficulties of students [39], the impact of the amount/type of visual material displayed in the classroom in visual cognition processes for DHH students, needs further research.

A growing body of research revealed underlying teachers’ beliefs on learning and our sample of teachers of DHH students are no exception. By our fourth research question, about brain-learning-related myths identification, we observed similar patterns of response as in previous studies [5,15,29], in which, for example, approximately 95% of the teachers agree with the learning styles practices [5].

When analysing the teaching experience time with DHH students, we partially confirm our hypothesis, since no significant differences were found in each country, but when we look at the total teachers’ sample, we found that more years of experience does not mean fewer beliefs in neuromyths. This joint sample difference corroborates previous studies that teachers in training showed fewer false beliefs than teachers who are already working [40]. One of the explanations may be related to the fact that teachers with more experience have been more exposed to sources of information, whether they are trustworthy or not, and can access the neuroscientific literature more lightly, as looking for very specific answers and over-interpreting results, which can easily lead to extrapolations to find pedagogical recipes. The Visual-Auditory-Kinesthetic (VAK) learning style is a widely cited example of misconceptions about the brain functioning where both old and recent research recognized it as a weak educational application [4]. It is also a good example of a false belief with a high percentage except if the sample is of trainee teachers [5,14,16,30,32,41]. So, informing teachers in the training process with reliable scientific sources and giving them the tools to be able to check data, could counteract the current trend in more experienced teachers [40,41].

Even though the main reasons are still under open discussion, common predictors for false beliefs may be due to teacher training in different cohorts or little scientific informed curriculum over training time. Several authors have been protesting about persistent myths, as VAK approach, with the aggravating factor that teachers continue to attend courses organized by their schools, and even after they are enlightened about the lack of evidence, they continue working under that perspective [42]. The persistence of neuromyths is sustained by specific cultural conditions, such as the spread of pseudoscience and the desire for exciting brain news [43]. As for the teachers’ interest in reading scientific literature and the impact on the detection of neuromyths, there is no difference between the two countries analysed (those who read, or not, scientific literature) in the total of false beliefs. However, previous research also suggests that greater general knowledge about the brain does not appear to protect teachers from picking up neuromyths [5,14,29]. When we analyse the responses of each sample separately, we can notice that in Portugal, those who state that read scientific literature, also give more wrong answers in the “second language ​​acquisition” myth. This represents important data to discuss in the field of deaf education, given the implications in the acceptance and pedagogical implementation of bilingual programs for DHH children. Regarding the Portuguese context, we consider the fact that much of the scientific literature available is disclosed in English which could discourage to some extent. To minimize the spread of these misconceptions among teachers, more outreach materials, textbooks for educators, or the popularization of science in schools are some examples to increase scientific literacy in teachers. We are currently seeing a widespread need in education to help teachers to be aware of the dangers of online misinformation, by recognizing more easily the good sources of information, and this is also extendable to special education domains. This issue is also in line with our last research question, in which the reported weak access to updated knowledge seems to be a key factor for the low levels of teachers’ confidence degrees about their practices, more markedly in Portuguese teachers. Also, other justifications for the pedagogical-didactic insecurities were found exclusively in the Portuguese sample. The presence of the sign language interpreter in the classroom was mentioned, since the fact that there is always someone mediating communication, by translating both teacher and student, and the extra “noise” from the primary transmission channel could misunderstand the teachers’ pedagogical choices. Data also shows that Portuguese teachers learned sign language later than Swedish teachers. We could discuss if teachers had earlier training in Portuguese Sign Language, they probably would develop more their communication skills with DHH students, feeling less "noise" in the communication, even when the sign interpreter is present in class. Another difference about the Portuguese teachers’ confidence degree reported is the concern with the students’ degrees of deafness, revealing that they feel less prepared with profound deafness students. Concerning the relationship that was observed between the teachers’ false beliefs and the agreement of enhanced visual abilities of DHH students, data reveal how the visual component is also misinterpreted concerning DHH people. The cognitive and brain sciences have been misused in which the neuromyths in education presented side effects.

Overall, although teachers’ false beliefs need to be worked on in both countries, it is in the Portuguese sample that we perceive the importance of up-to-date teacher training to increase the self-confidence in pedagogical decisions with DHH students and to conduct the best educational practices based on science.

## Conclusions

Based on the teachers’ perceptions, the main contribution of the study was to present a more comprehensive picture of how the learning of DHH students is described, by unveiling parallelisms between Portuguese and Swedish teachers’ beliefs.

The perceptions of schoolteachers coincide at the academic achievement level, well below desirable, mainly in ​​the language school domain. Portuguese teachers tend to attribute the DHH students’ attention difficulties to their idiosyncrasies (such as visual acuity, degree of interest, or deafness), reinforcing erroneous perceptions about their educational needs and potentials to achieve school success. However, our survey showed conceptions were revealed in which teachers believe they can intervene, and these are the ones that can change pedagogical and didactic options and benefit access of DHH students to *curricula*. Controlling visual stimuli in the classroom to manage students’ effort in attention tasks is one of the factors reported. Despite we have gathered positive indicators of teachers’ concerns with learning challenges, our results are in line with the broad literature that identifies misconceptions in education. Portuguese and Swedish teachers shared beliefs in neuromyths with a prevalence of preferred learning style, also for DHH students. Even the most experienced teachers, being a common feature verified in both countries, succumb to the learning myths presented. Evidence-based practice is needed as it recognizes factors from pedagogical-didactic nature, that can, for example, prevent unnecessary levels of fatigue that lead to cognitive overload in DHH students and consequent failure rate at school. What our study stresses are that the visual cognition premise in DHH learners can be wrongly perceived, and simultaneously creating an illusion of attendance to the students’ educational needs [11]. Changes in special education are claimed by teachers and, according to our results, these can involve more teacher training and partnerships between researchers and teachers based on updates coming from the cognitive neurosciences.

## Limitations of the study

We acknowledge the impossibility of getting a more in-depth knowledge of both educational contexts’ characteristics. If on the one hand, samples belong to realities with some common ground in education foundation for the deaf students, on the other, both countries follow now different ways in their educational systems (i.e., teacher´s training programs, the possibility of DHH education in mainstream schools/ special schools and conflicting arguments around the inclusive educational paradigm for DHH students). Although we did not have enough data to clarify all these possible differences, this is in some way reflected in the scientific literature of both countries, which seems to serve as a reference on the teacher’s practice [12,19,21,25,26,29,37,40]. Portuguese sample is the more aged one, but do not have the highest percentage in terms of experience working with DHH students. By coinciding age range and the work experience variables, there is a probability that the Portuguese sample was more influenced by demand effects in filling out the questionnaire, trying to anticipate the purposes of the study to respond in an "appropriate manner”, generating a bigger probability of bias occurrence in the collected data. We realize that a self-report survey, even if anonymous and online, runs the risk of responses based on social desirability. Another limitation concerns our difficulties to obtain a larger sample and estimating representative population size in both countries.

## Future research and educational implications of findings

Further studies aim at collecting more specific information on the effective use of visual-orientated strategies as to how to better attend visual cognition (i.e., typology of visual resources, images display criteria in a classroom, etc.). Doing so would bring important additional inputs to the research with deaf students and their teaching. Conduct interdisciplinary research, by combining the field of deaf studies and Neuroscience, towards the effectiveness of teaching for DHH students, is also increasingly required to increase scientific literacy capable of reducing neuromyths in education. This study also contributes to highlighting the future need for translation of scientific knowledge directed to the school’s interests.

## Supporting information

S1 Fig Comparing teachers’ perceptions about the language performance levels of DHH students (N = 133). (TIF)

S2 Fig Comparing teachers’ perceptions about the mathematics performance levels of DHH students (N = 133). (TIF)

S3 Fig Comparing teachers´ perceptions about DHH students’ visual cognition skills (N = 133). (TIF)

S4 Fig Comparing teachers’ perception about increased visual skills manifestation ages (N = 133). (TIF)

S5 Fig Comparing teachers’ conceptions about DHH learners’ difficulties in maintaining attention in class (N = 133). (TIF)

S6 Fig Comparing teachers’ conceptions about the importance gave to visual material exhibition in classroom walls (N = 133). (TIF)

S7 Fig Teacher’s confidence comparison in Pedagogical options (N = 133). (TIF)

S8 Fig Compared teachers preferred training modalities (N = 133). (TIF)

S1 Table Demographic data of Portuguese and Swedish teachers. (TIF)

S2 Table Comparing Portuguese and Swedish teachers’ answers concerning myth/fact statements (N = 133). ^a^ In this table the p-values are NS (or p> 0.05).
(TIF)

S3 Table Data association between teachers’ answers (question about DHH visual belief crossed with learning neuromyth classification) (N = 133). (TIF)

S1 File Portuguese version of the survey questionnaire: Conceções e Práticas de professores e educadores de infância acerca dos estilos de aprendizagem dos alunos surdos. (PDF)

S2 File Swedish version of the survey questionnaire: Lärares uppfaning om inlärningssä bland elever med hörselnedsäning I grundskolan. (PDF)
