# Supplementary figures and images for: Unveiling teachers’ beliefs on visual cognition and learning styles of deaf and hard of hearing students: A Portuguese-Swedish study
Source: PLoS One. 2022 Feb 15;17(2):e0263216. doi: 10.1371/journal.pone.0263216 (PMC9116990; doi:10.1371/journal.pone.0263216)

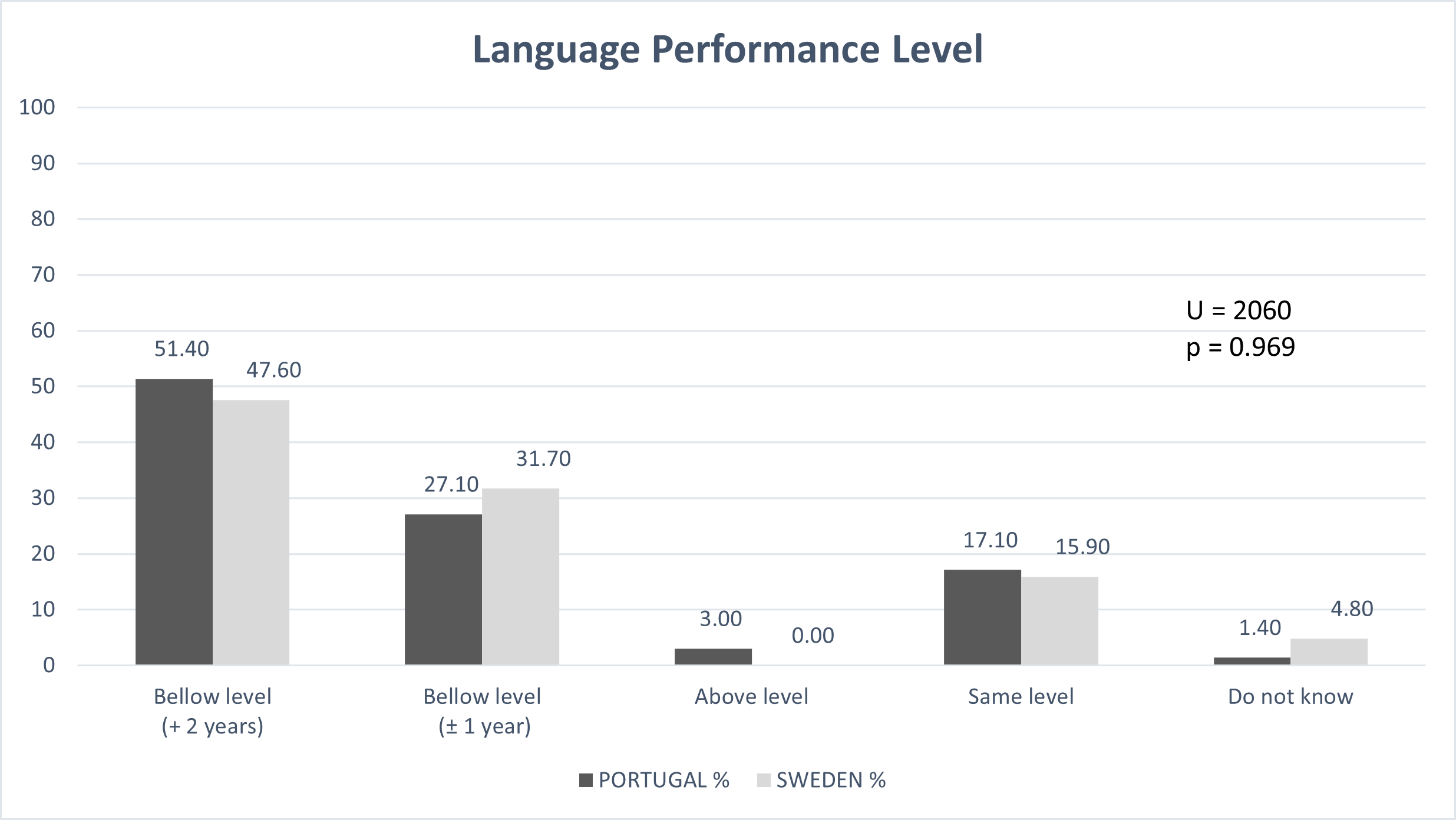

Supplement: S1 Fig — (TIF) [file pone.0263216.s001.tif]

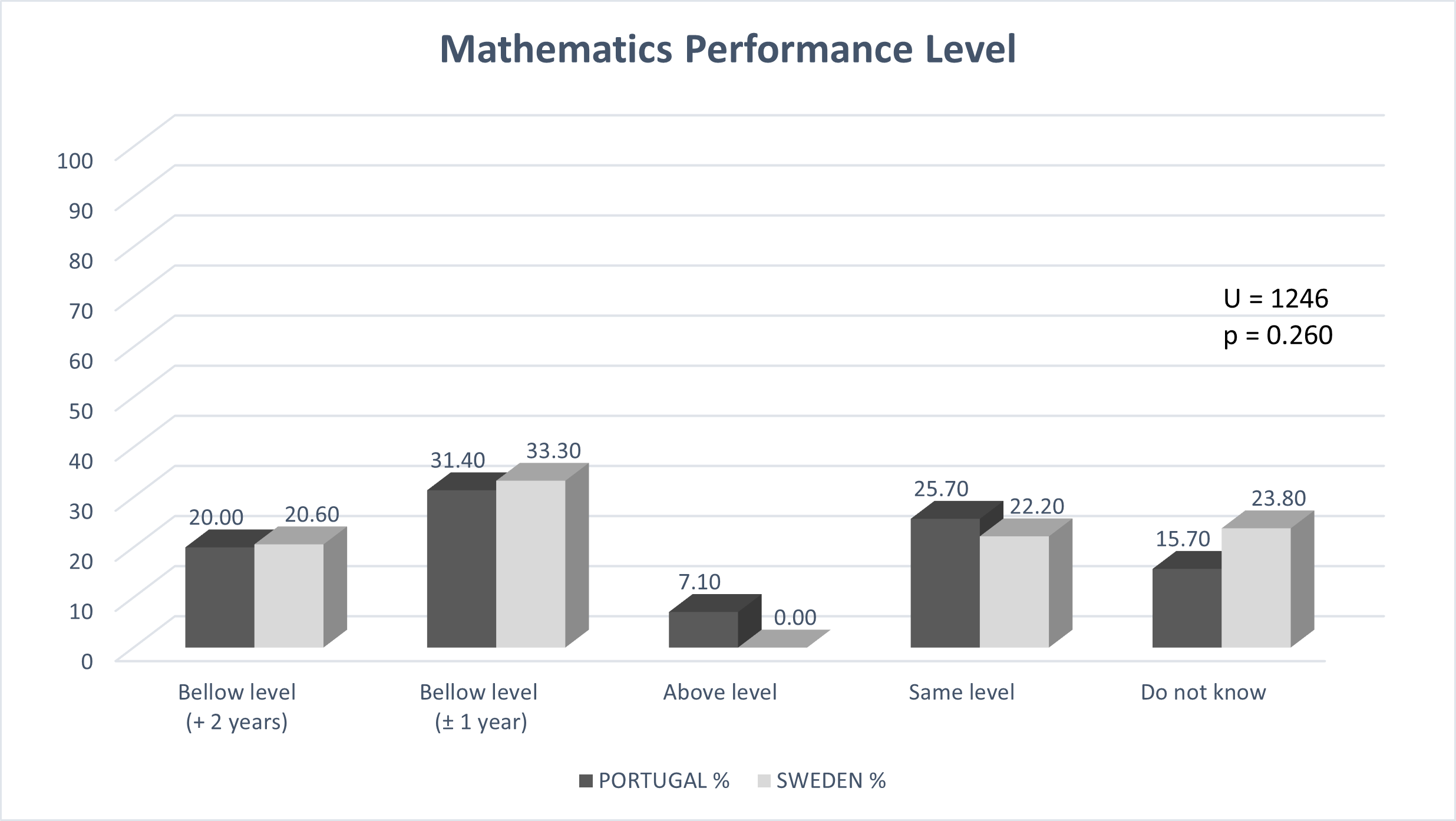

Supplement: S2 Fig — (TIF) [file pone.0263216.s002.tif]

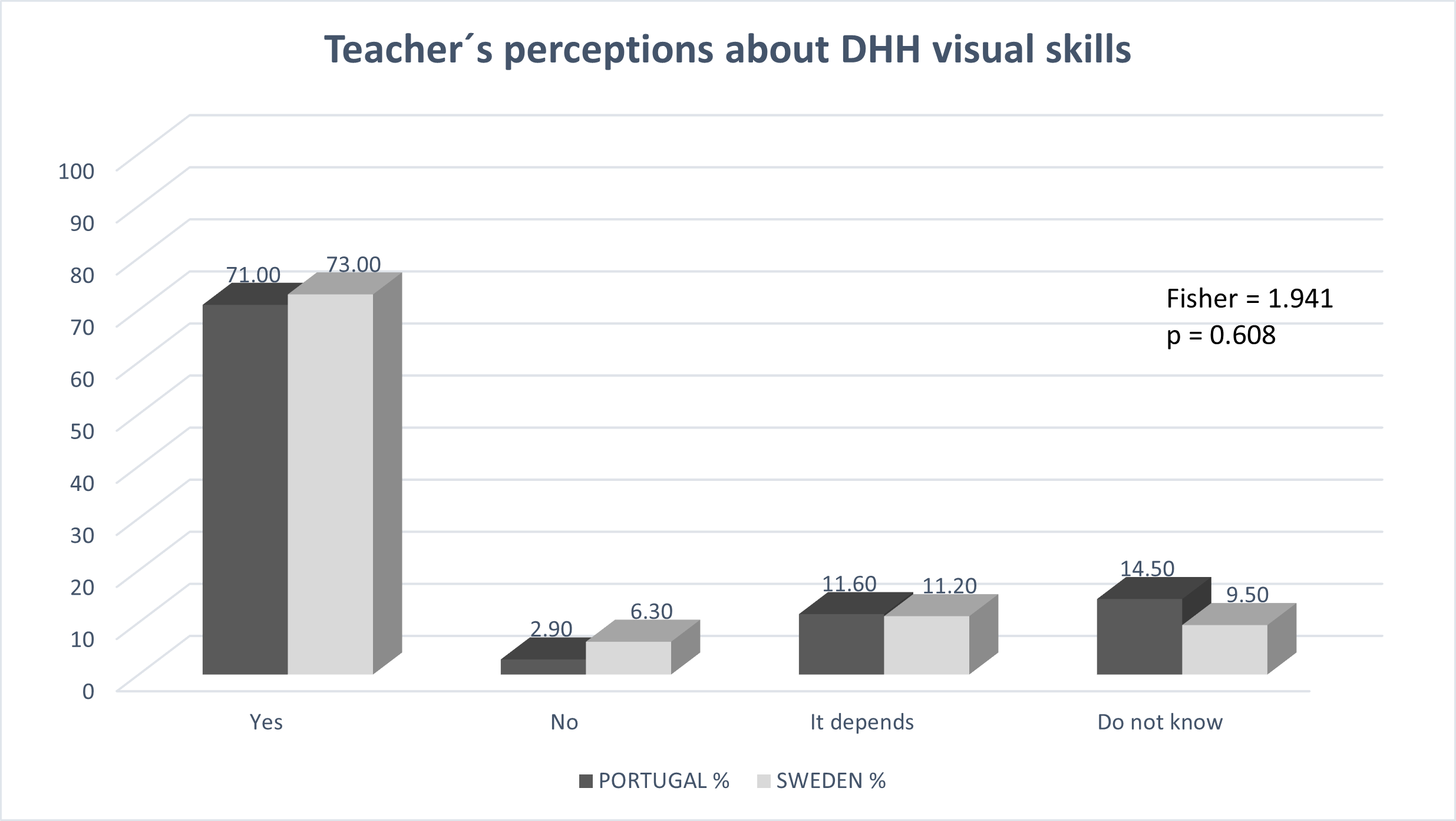

Supplement: S3 Fig — (TIF) [file pone.0263216.s003.tif]

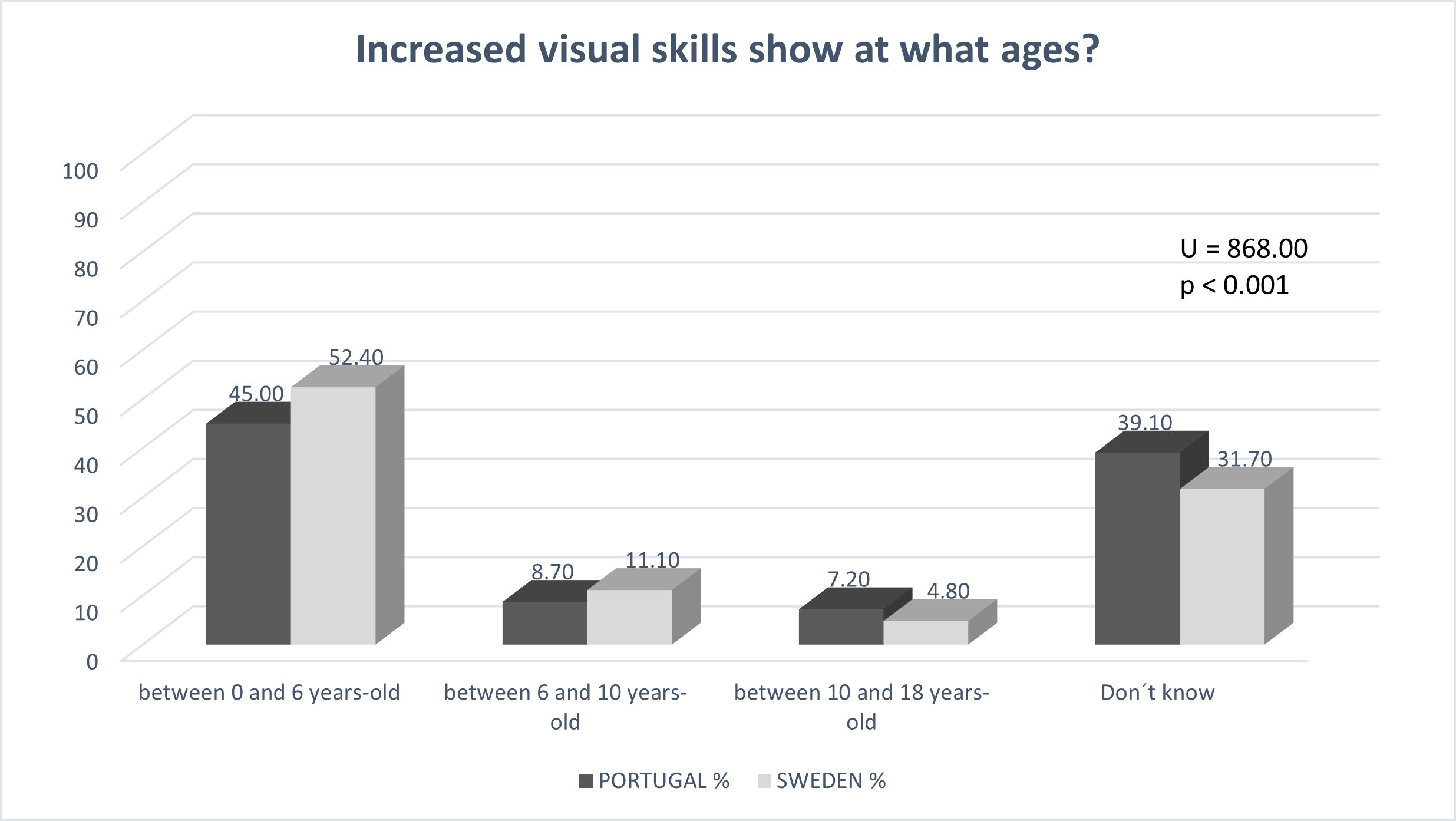

Supplement: S4 Fig — (TIF) [file pone.0263216.s004.tif]

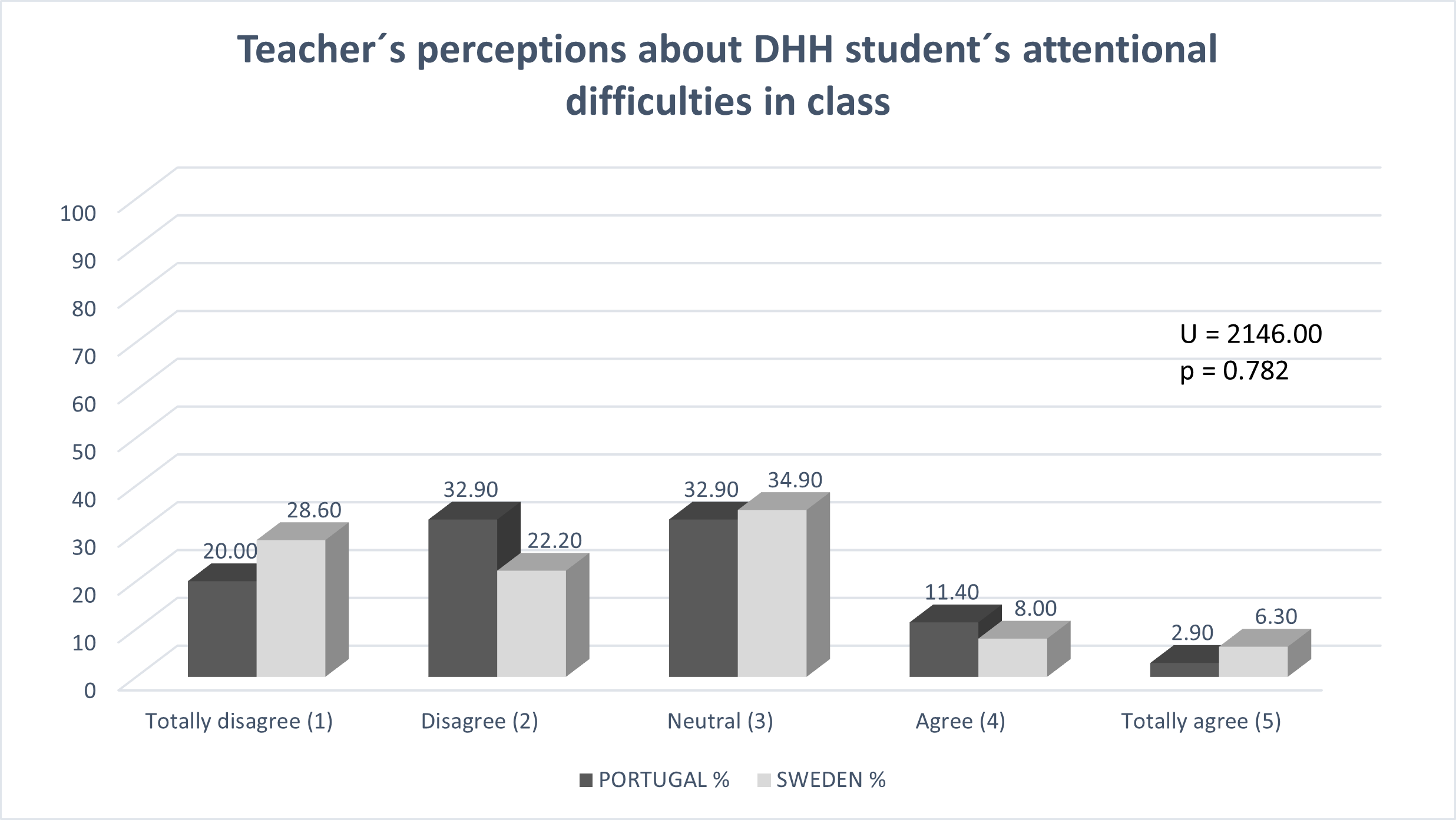

Supplement: S5 Fig — (TIF) [file pone.0263216.s005.tif]

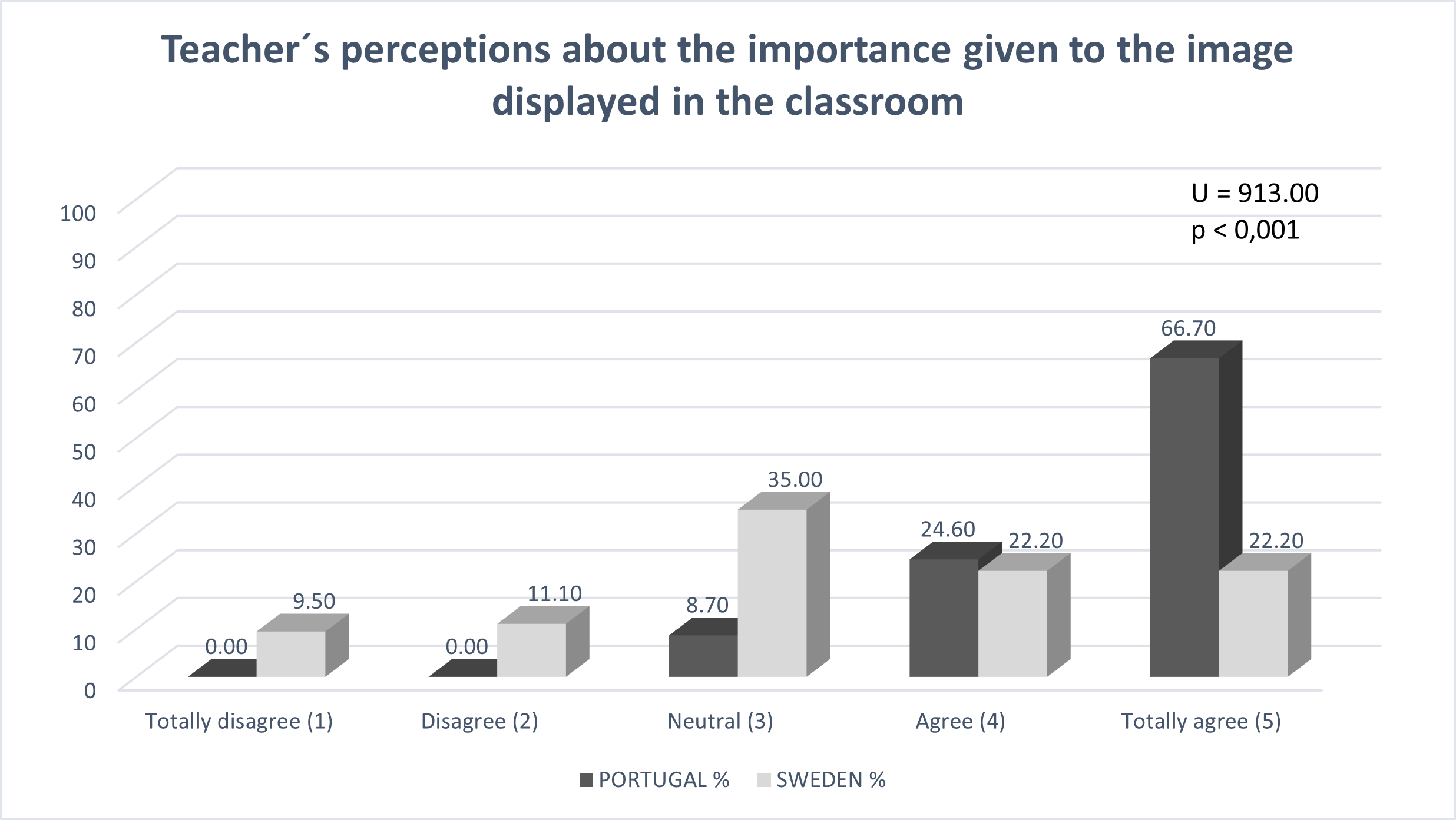

Supplement: S6 Fig — (TIF) [file pone.0263216.s006.tif]

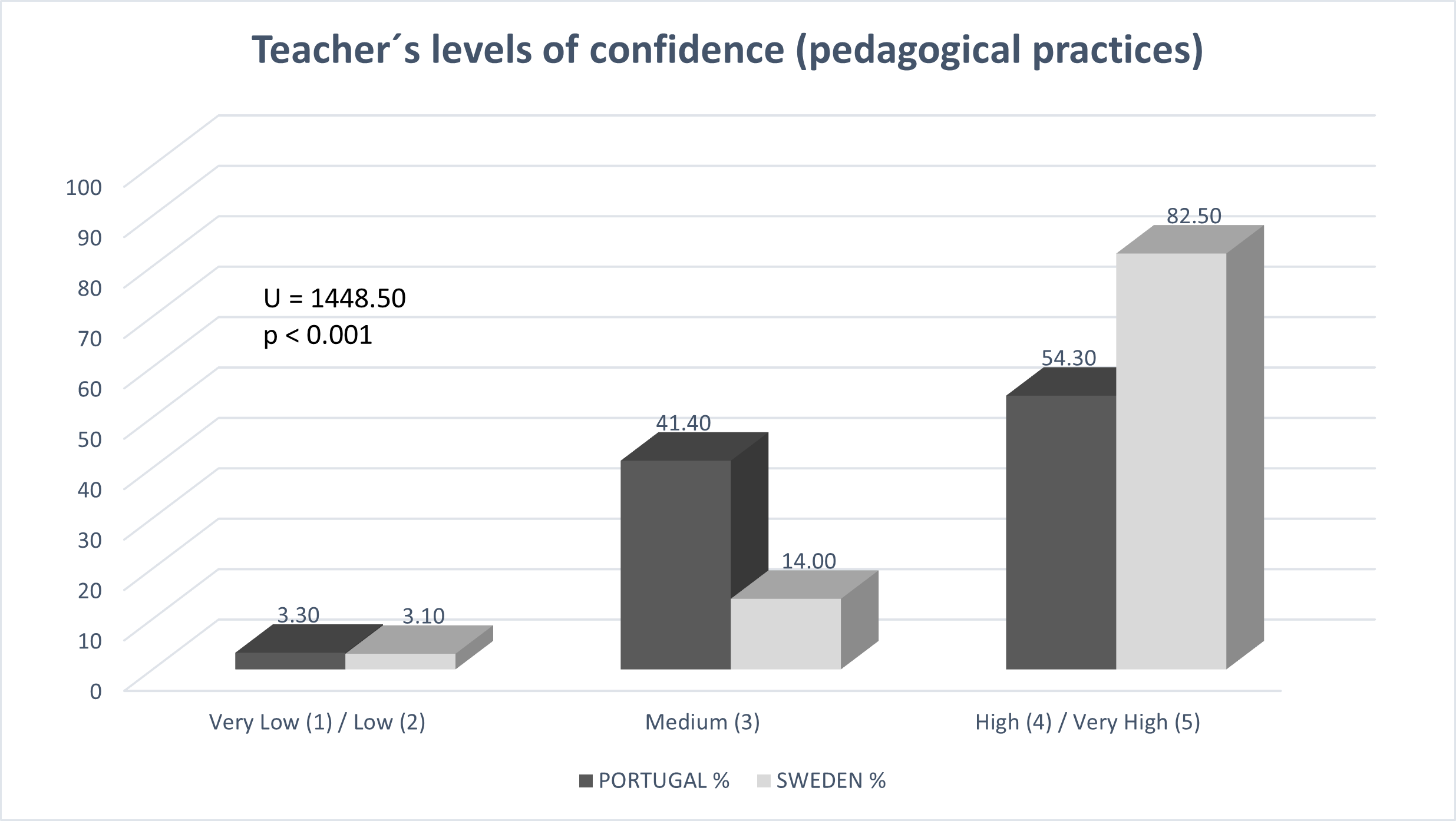

Supplement: S7 Fig — (TIF) [file pone.0263216.s007.tif]

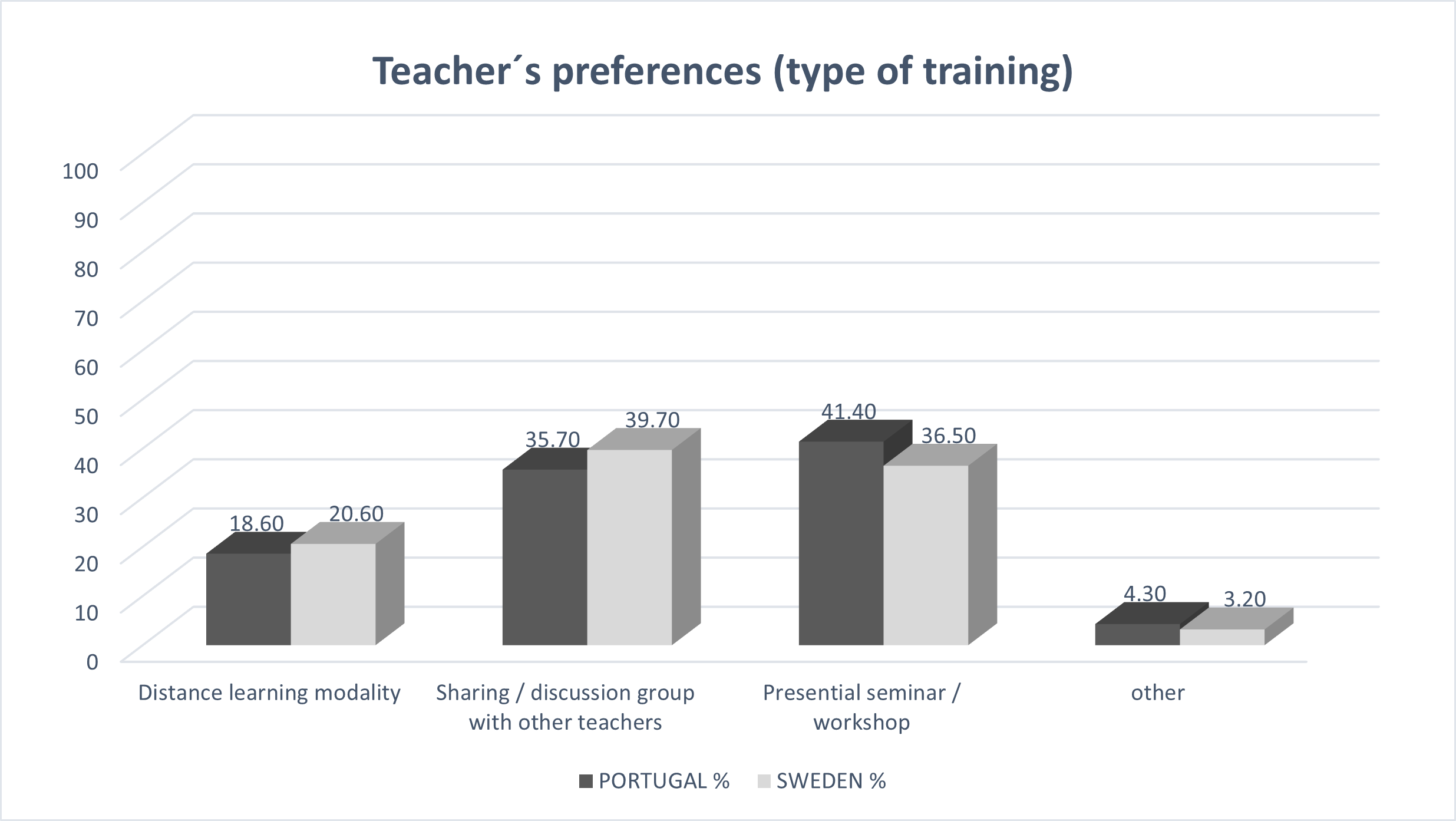

Supplement: S8 Fig — (TIF) [file pone.0263216.s008.tif]

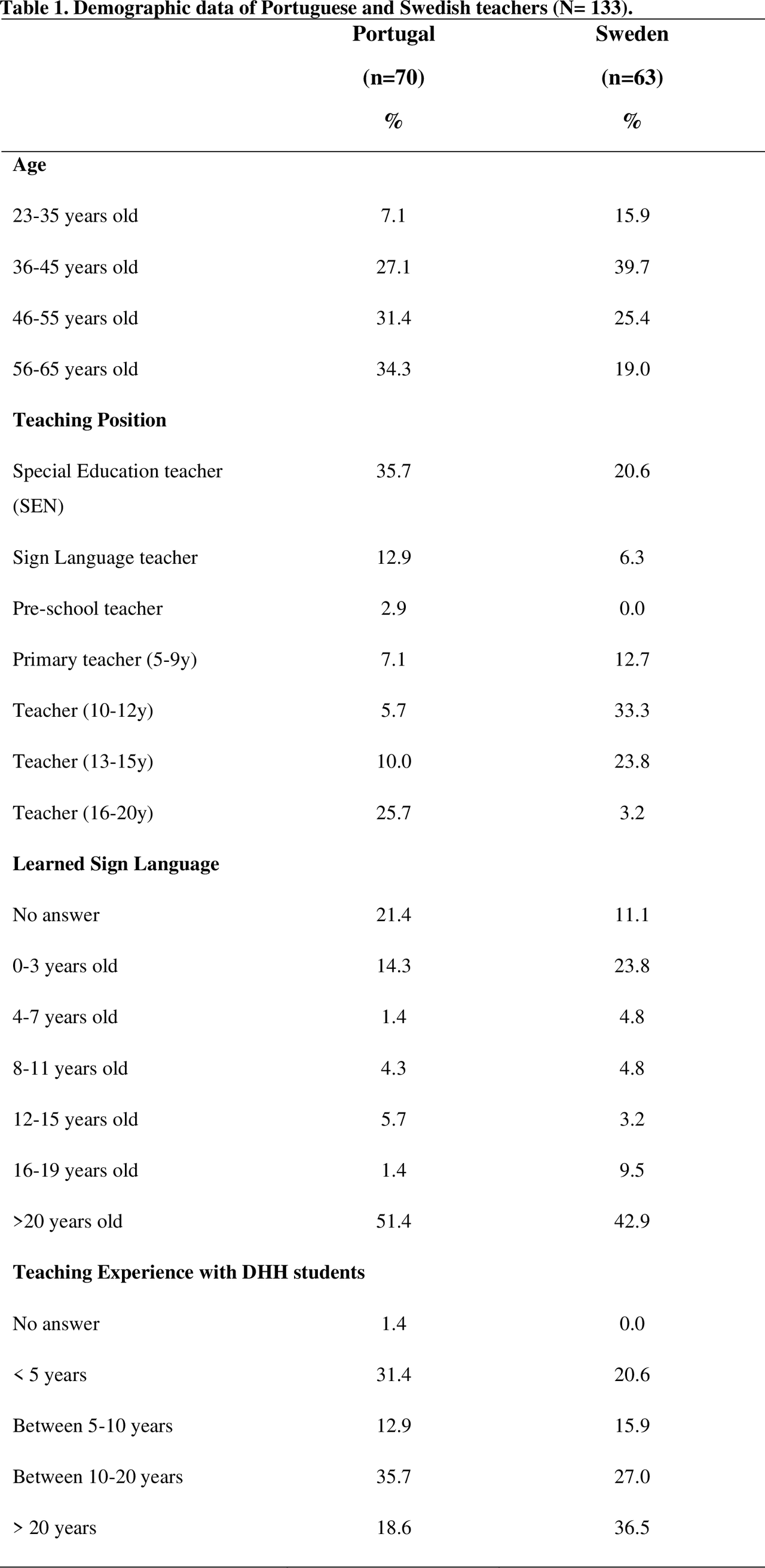

Supplement: S1 Table — (TIF) [file pone.0263216.s009.tif]

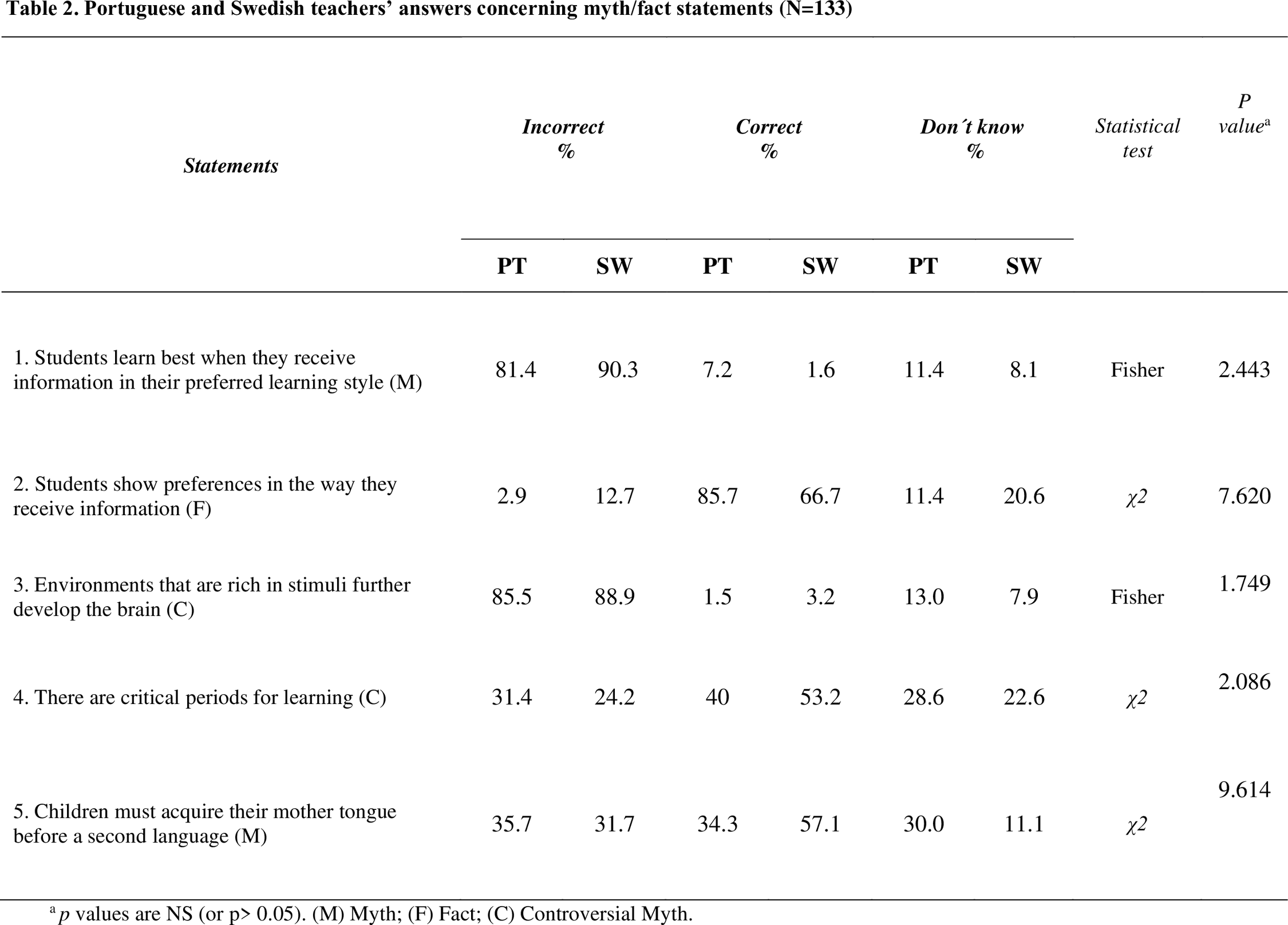

Supplement: S2 Table — a In this table the p-values are NS (or p> 0.05). (TIF) [file pone.0263216.s010.tif]

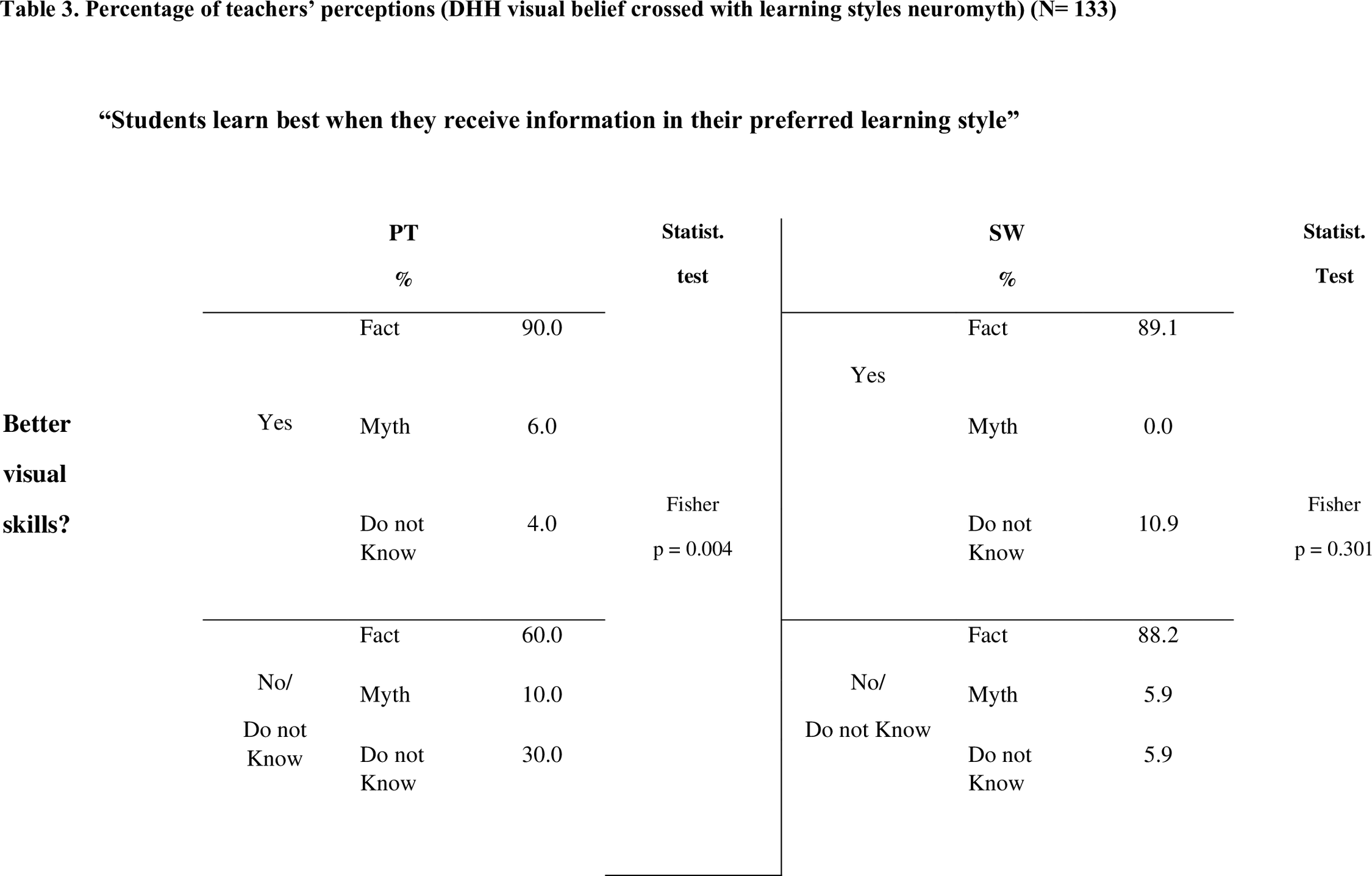

Supplement: S3 Table — (TIF) [file pone.0263216.s011.tif]
