# Supplementary material for: Unveiling teachers’ beliefs on visual cognition and learning styles of deaf and hard of hearing students: A Portuguese-Swedish study
Source: PLoS One. 2022 Feb 15;17(2):e0263216. doi: 10.1371/journal.pone.0263216 (PMC9116990; doi:10.1371/journal.pone.0263216)
Supplement: S1 File — (PDF) [file pone.0263216.s012.pdf]

Conceções e práticas de professores e educadores de infância acerca dos estilos de aprendizagem dos alunos surdos

Prezado(a) Professor(a)/ Educador(a):
O presente questionário enquadra-se no âmbito de um programa doutoral desenvolvido na área de Educação para Surdos, realizado no Instituto de Ciências da Saúde (ICS), Universidade Católica Portuguesa em parceria com a Universidade de Estocolmo.
Neste sentido, pedimos a sua colaboração para o preenchimento do documento apresentado. Importa referir que o questionário é anónimo e os dados serão utilizados apenas no âmbito da investigação em questão.

Para responder a este questionário por favor considere apenas as crianças ou alunos com surdez e sem outras patologias ou perturbações sensoriais ou cognitivas, a frequentar o ensino obrigatório.

Perfil do inquirido

1. 1. Idade

   Marcar apenas uma oval.

   - entre 25 e 35 anos
   - entre 36 e 45 anos
   - entre 46 e 55 anos
   - entre 56 e 65 anos
   - outro

2. 2. Habilitações académicas

Marcar apenas uma oval.

- Licenciatura
- Pós-graduação / especialização
- Mestrado
- Doutoramento
- Outra

3. 3. País - Portugal (zona onde trabalha)

Marcar apenas uma oval.

- Norte
- Centro
- Sul

4. 4. Consulta habitualmente literatura científica nas áreas da Educação, Surdez ou das Neurociências?

Marcar apenas uma oval.

- sim
- não
- não respondo

5. 5. Em que contexto leciona ou já lecionou/ presta ou prestou apoio pedagógico a alunos surdos

Marcar apenas uma oval.

- Contexto Português - num Agrupamento/ Escola de Referência para Ensino Bilingue de Alunos Surdos (EREBAS)
- Contexto Português - num Agrupamento sem Ensino Bilingue de Alunos Surdos
- Outra

6. 6. Cargo que ocupa /ocupou no Agrupamento

Marcar apenas uma oval.

- Educador(a) Creche
- Educador(a)(Pré-escolar-JI)
- Professor(a) do 1º ciclo
- Professor do 2º ciclo
- Professor(a) do 3º ciclo
- Professor(a) do ensino secundário
- Docente de Educação Especial (grupo 920)
- Docente de Língua Gestual

7. 7. Organização da turma na qual leciona ou presta apoio (ex: turma de surdos ou turma de surdos integrados)

____

8. 8. Perfil auditivo - que opção o/a caracteriza melhor ?

Marcar apenas uma oval.

- Pessoa Surda
- Pessoa com Surdez
- Ouvinte
- Não respondo

9. 9. Com que idade aprendeu Língua Gestual Portuguesa?

____

10. 10. Utiliza a Língua Gestual Portuguesa ou Português gestualizado com os alunos a quem leciona?

Marcar apenas uma oval.

- Sim
- Não

11. 11. Experiência de ensino com alunos surdos

Marcar apenas uma oval.

- até 5 anos
- entre 5 e 10 anos
- entre 10 a 20 anos
- mais de 20 anos

12. 12. No grupo de crianças/ alunos surdos a que leciona /lecionou, a maioria normalmente...

Marcar apenas uma oval.

- Compreendem e expressam ideias através da oralidade
- Compreendem e expressam ideias através da LGP
- Compreendem e expressam ideias usando quer a oralidade quer a LGP

13. 13. Na sua opinião, em que nível se encontram (ou encontravam) os alunos em Língua Portuguesa quando comparados com os pares ouvintes?

Marcar apenas uma oval.

- Acima do nível de performance
- No mesmo nível de performance
- Abaixo do nível de escolaridade (± 1 ano)
- Abaixo do nível de escolaridade (+ de 2 anos)

14. 14. E em Matemática?

Marcar apenas uma oval.

- Acima do nível de performance
- No mesmo nível de performance
- Abaixo do nível de escolaridade (± 1 ano)
- Abaixo do nível de escolaridade (+ de 2 anos)

Segunda secção / sektion 2 ESTILOS DE APRENDIZAGEM – Responda de acordo com a sua opinião pessoal

15. 15. De acordo com a sua conceção, comparativamente aos alunos ouvintes na mesma faixa etária, é provável que as crianças/ os alunos surdos possuam melhores capacidades ao nível Visual (ex.: perceção visual, atenção visual e memória visual)?

Marcar apenas uma oval.

- sim
- não
- não sei
- depende (se considera que a existência destas capacidades visuais depende de algum fator em particular, por favor explicite qual a seguir

16. 15.1. Considero que depende de factores como:

17. 16. Este ganho de visualidade nas crianças com surdez poderá ser manifestado...

Marcar apenas uma oval.

- Entre os 0 e 6 anos de idade
- Entre os 6 e os 10 anos de idade
- Entre os 10 e 18 anos de idade
- A partir dos 18/anos de idade
- Não sei

18. 17. Por serem "aprendentes visuais" , beneficiam se as estratégias de ensino forem mais baseadas em imagens e em pistas visuais.

Marcar apenas uma oval.

	1	2	3	4	5	
discordo completamente						concordo completamente

19. 18. A exposição de um conjunto diversificado de imagens nas paredes da sala de aula de 1º ciclo ou JI onde existem crianças/ alunos surdos, é importante para reforçar as suas aprendizagens.

Marcar apenas uma oval.

	1	2	3	4	5	
discordo completamente						concordo completamente

20. 19. As imagens que são mediadas pela ação do/a professor/a têm uma influência superior nas aprendizagens dos alunos surdos do que as imagens apresentadas de forma menos dinâmica?

Marcar apenas uma oval.

	1	2	3	4	5	
discordo completamente	◯	◯	◯	◯	◯	concordo completamente

21. 20. Na sua opinião, as crianças/ os alunos surdos têm mais dificuldades em manter a atenção visual do que os seus pares ouvintes.

Marcar apenas uma oval.

	1	2	3	4	5	
discordo completamente	◯	◯	◯	◯	◯	concordo completamente

22. 20.1 Por favor explique porquê

____
____
____
____
____

23. 21. Na sua opinião, a razão pela qual a criança / o aluno surdo possa descodificar melhor a informação quando esta é apresentada através de imagens, relaciona-se com a natureza visioespacial e cinética da língua gestual.

Marcar apenas uma oval.

	1	2	3	4	5	
discordo completamente	◯	◯	◯	◯	◯	concordo completamente

24. 22. Do conhecimento que tem sobre a pesquisa científica no campo da Educação para Surdos, quais os métodos considerados mais eficazes no processo de ensino e aprendizagem da leitura e escrita da língua portuguesa?

Marcar apenas uma oval.

- os métodos visuais que partem globalmente do texto/frases para as unidades mais pequenas- letra (Globais ou Analíticos)
- os métodos fonéticos que partem dos fonemas e grafemas para a palavra - frase-texto (Sintéticos)
- os métodos mistos (combinação de métodos Analíticos-Sintéticos)
- não sei
- outros

25. 22.1 Se respondeu «Outros», por favor explicite

26. 23. Na área da matemática, os conceitos devem ser complementados com algum tipo de suporte visual (esquemas, gráficos, diagramas, etc.), pois a informação dada nos enunciados verbais é insuficiente para o processo de raciocínio do aluno surdo.

Marcar apenas uma oval.

	1	2	3	4	5	
discordo completamente						concordo completamente

27. 23.1 Por favor, explique o porquê da sua resposta anterior

Terceira secção - Neurociência e Educação / Por favor escolha entre as opções MITO, FACTO ou NÃO SEI

28. 24. Os alunos aprendem melhor quando recebem as informações no seu estilo de aprendizagem preferencial (ex.: estilo auditivo, visual, cinestésico).

Marcar apenas uma oval.

- Facto
- Mito
- Não sei

29. 25. Os alunos mostram preferências no modo em que recebem informações (ex.: auditivo, visual, cinestésico).

Marcar apenas uma oval.

- Facto
- Mito
- Não sei

30. 26. Os ambientes que são ricos em estímulos desenvolvem mais o cérebro da criança em idade pré-escolar.

Marcar apenas uma oval.

- Facto
- Mito
- Não sei

31. 27. Existem períodos críticos na infância a partir dos quais certas coisas deixam de poder ser aprendidas.

Marcar apenas uma oval.

- Facto
- Mito
- Não sei

32. 28. As crianças devem adquirir a sua língua materna antes de uma segunda língua ser aprendida. Caso contrário, nenhuma das línguas será totalmente adquirida.

Marcar apenas uma oval.

- Facto
- Mito
- Não sei

33. 29. Devido a uma maior dependência da informação visual para conhecer e interpretar o mundo, o aluno surdo deverá ter a oportunidade de, na escola, adquirir mais competências relacionadas com a eficácia da sua visualidade e literacia visual.

Marcar apenas uma oval.

- sim
- não
- não sei

34. 30. Sente-se confiante quanto à seleção de metodologias /estratégias pedagógicas e didáticas que aplica junto dos alunos surdos do seu grupo/ turma? (escolha um valor de 1 a 5).

Marcar apenas uma oval.

	1	2	3	4	5	
nada confiante	◯	◯	◯	◯	◯	totalmente confiante

35. 30.1 Gostaríamos que comentasse o porquê da sua resposta

36. 31. Se sente necessidade de atualizar o conhecimento científico-pedagógico acerca da Educação para Surdos, em qual das modalidades pensa que poderia, eventualmente, vir a participar?

Marcar apenas uma oval.

- ◯ Ação de formação na modalidade de ensino à distância
- ◯ Seminário/ workshop presencial
- ◯ Grupo de partilha /discussão com outros professores
- ◯ outra

Muito obrigados pela sua participação.

Este conteúdo não foi criado nem aprovado pela Google.

Google Formulários
