# Supplementary material for: Unveiling teachers’ beliefs on visual cognition and learning styles of deaf and hard of hearing students: A Portuguese-Swedish study
Source: PLoS One. 2022 Feb 15;17(2):e0263216. doi: 10.1371/journal.pone.0263216 (PMC9116990; doi:10.1371/journal.pone.0263216)
Supplement: S2 File — (PDF) [file pone.0263216.s013.pdf]

Lärares uppfattning om inlärningssätt bland elever med hörselnedsättning i grundskolan

Till dig som undervisar döva eller hörselskadade elever:
Denna enkätundersökning genomförs inom ramen för ett doktorandprojekt som hör till området "Dövundervisning", vid Institute of Health Sciences (ICS), Universidade Católica Portuguesa i Portugal. Vi vill be dig att bidra till projektet genom att besvara enkäten. Ditt svar är helt anonymt och uppgifterna kommer endast att användas inom ramen för det här projektet.

När du svarar på frågorna ska du bara fokusera på grundskoleelever som har någon form av hörselnedsättning och som inte har några andra sensoriska eller kognitiva funktionsnedsättningar.

Pedagogens bakgrund och erfarenhet

1. 1. ålder - jag är

Marcar apenas uma oval.

- mellan 25 och 35 år
- mellan 36 och 45 år
- mellan 46 och 55 år
- mellan 56 och 65 år
- annan ålder

2. 2. Akademiska kvalifikationer

Marcar apenas uma oval.

- Lärarexamen eller förskollärarexamen
- Speciallärarexamen eller specialpedagogexamen
- Magister eller masterexamen
- Doktorsexamen
- Annan

3. 3. Region

Marcar apenas uma oval.

- Götaland
- Svealand
- Norrland

4. 4. Brukar du konsultera vetenskaplig litteratur inom områdena dövundervisning, dövstudier eller neurovetenskap?

Marcar apenas uma oval.

- ja
- nej
- inget svar

5. 5. Inom vilken utbildningskontext undervisar/stöttar du elever med hörselnedsättning?

Marcar apenas uma oval.

- Inom vanlig förskola/grundskola
- Inom specialskolan
- Annan

6. 6. Anställd som

Marcar apenas uma oval.

- Förskollärare (ålder ± 1 till 5)
- Lärare i förskoleklass/på lågstadium (6-9 år)
- Lärare på mellanstadium (10-12 år)
- Lärare på högstadium (13-15 år)
- Lärare på gymnasieskola (16-20 år)
- Speciallärare/specialpedagog
- Teckenspråkslärare

7. 7. Hur är undervisningen organiserad (t.ex. i form av dövklass, hörselklass eller individuell placering med hörande klasskamrater)?

8. 8. Vilken är din egen hörselstatus?

Marcar apenas uma oval.

- döv
- hörselskadad
- hörande
- inget svar

9. 9. Vid vilken ålder lärde du dig svenskt teckenspråk?

10. 10. Använder du svenskt teckenspråk eller någon form av tecken som stöd med de elever du undervisar?

Marcar apenas uma oval.

- Ja
- Nej

11. 11. Antal år med erfarenhet av elever med hörselnedsättning

Marcar apenas uma oval.

- mindre än 5 år
- mellan 5 och 10 år
- mellan 10 och 20 år
- mer än 20 år

12. 12. De flesta av dina elever med hörselnedsättning ...

Marcar apenas uma oval.

- förstår och uttrycker sig huvudsakligen genom att tala och lyssna
- förstår och uttrycker sig huvudsakligen genom svenskt teckenspråk
- förstår och uttrycker sig huvudsakligen genom att tala och teckna samtidigt

13. 13. Vilken nivå bedömer du att eleverna har i svenska i jämförelse med jämnåriga?

Marcar apenas uma oval.

- över
- på samma nivå
- under (± 1 år)
- mycket under (mer än 2 år)
- vet inte

14. 14. Vilken nivå bedömer du att eleverna ligger på i matematik i jämförelse med jämnåriga?

Marcar apenas uma oval.

- över
- på samma nivå
- under (± 1 år)
- mycket under (mer än 2 år)
- vet inte

Sektion 2 LÄRANDESTILAR - Svara utifrån din egen personliga uppfattning

15. 15. Har elever med hörselnedsättning bättre visuella färdigheter (t.ex. visuell uppfattning, visuell uppmärksamhet och visuellt minne) jämfört med hörande elever?

Marcar apenas uma oval.

- ja
- nej
- vet inte
- det beror på / Om du anser att förekomsten av dessa visuella förmågor beror på vissa faktorer, förklara i fältet nedan

16. 15.1. Jag tror att detta har att göra med faktorer som:

17. 16. Vinsterna med ökad visuell förmåga uppstår när barnet är ...

Marcar apenas uma oval.

- mellan 0 och 6 år gammal
- mellan 6 och 10 år gammal
- mellan 10 och 18 år gammal
- från 18 års ålder
- vet inte

18. 17. Genom att vara "visuella inlärare" drar eleverna nytta av undervisningsstrategier som i högre grad bygger på bilder och visuella beståndsdelar.

Marcar apenas uma oval.

	1	2	3	4	5	
håller inte alls med						håller fullständigt med

19. 18. Det är viktigt att elever med hörselnedsättning exponeras för en varierande mängd bilder som finns uppsatta på klassrummets väggar

Marcar apenas uma oval.

	1	2	3	4	5	
håller inte alls med						håller fullständigt med

20. 19. Bilder som används och medieras av lärare har större inflytande på studenters lärande än bilder som står för sig själva.

Marcar apenas uma oval.

	1	2	3	4	5	
Håller inte alls med						Håller fullständigt med

21. 20. Har elever med hörselnedsättning svårare att upprätthålla visuell uppmärksamhet än sina hörande jämnåriga?

Marcar apenas uma oval.

	1	2	3	4	5	
Nej	◯	◯	◯	◯	◯	Ja

22. 20.1 Berätta varför du tycker så

____
____
____
____
____

23. 21. Är anledningen till att elever med hörselnedsättning tycks vara bättre på att avkoda informationen när de får den genom bilder relaterad till teckenspråkets visuella-spatiala och kinestetiska natur?

Marcar apenas uma oval.

	1	2	3	4	5	
Nej, håller inte alls med	◯	◯	◯	◯	◯	Ja, håller fullständigt med

24. 22. Mot bakgrund av den vetenskapligt baserade kunskap du har om elever med hörselnedsättning, vilka metoder kan anses vara mest effektiva i undervisningen och inlärningsprocessen avseende att lära sig läsa och skriva svenska?

Marcar apenas uma oval.

- ( ) Analytiska metoder som går från helheten till delarna, dvs. man använder en top-down strategi som utgår från texten som helhet och slutar med igenkänning av bokstäverna.
- ( ) Syntetiska metoder som går från delarna till helheten, dvs. man använder en bottom-up strategi som utgår från bokstäverna eller språkljuden och går vidare mot ord, meningar och texter.
- ( ) En kombination av ovanstående metoder
- ( ) vet inte
- ( ) annat

25. 22.1 Om du valt «annat», förklara detta här

____

____

____

____

____

26. 23. Inom området matematik bör man stötta förståelsen av begrepp genom visuellt stöd (bilder, diagram, grafer) eftersom den information som ges i form av verbala påståenden inte passar problemlösningsprocessen hos elever med hörselnedsättning.

Marcar apenas uma oval.

	1	2	3	4	5	
Håller inte alls med	( )	( )	( )	( )	( )	Håller fullständigt med

27. 23.1 Vänligen, förklara hur du tänker angående detta

Del 3 - Neurovetenskap och utbildning: Vänligen välj mellan sant, falskt och vet inte

28. 24. Under skoltiden lär sig eleverna bäst om de får ta emot information genom det inlärningssätt de föredrar (t.ex. verbalt/auditivt, visuellt, kinestetiskt).

Marcar apenas uma oval.

- sant
- falskt
- vet inte

29. 25. Eleverna visar på vilket sätt de föredrar att få information (t.ex. verbalt/auditivt, visuellt, kinestetiskt).

Marcar apenas uma oval.

- sant
- falskt
- vet inte

30. 26. En stimulerande omgivning förbättrar förskolebarns hjärnor.

Marcar apenas uma oval.

- sant
- falskt
- vet inte

31. 27. Det finns kritiska perioder i barndomen efter vilka vissa saker inte längre kan läras in.

Marcar apenas uma oval.

- sant
- falskt
- vet inte

32. 28. Barn behöver tillägna sig sitt modersmål innan de börjar lära sig ett andraspråk. Om de inte gör det, så kommer de inte att kunna tillägna sig något av språken fullt ut.

Marcar apenas uma oval.

- sant
- falskt
- vet inte

33. 29. Eftersom döva och hörselskadade elever i högre grad är beroende av visuell information behöver de ges möjlighet att utveckla denna visualitet i skolan, liksom sina visuellt baserade läs- och skrivfärdigheter.

Marcar apenas uma oval.

- ja
- nej
- vet ej

34. 30. Är du trygg i ditt val av pedagogiska och didaktiska metoder/strategier som du tillämpar på elever med hörselnedsättning i din grupp/ klass (välj ett tal från 1 till 5)

Marcar apenas uma oval.

	1	2	3	4	5	
inte alls säker	( )	( )	( )	( )	( )	fullständigt säker

35. 30.1 Vänligen, kommentera ditt svar.

36. 31. Om du skulle känna behov av att uppdatera dina vetenskapliga och pedagogiska kunskaper som handlar om utbildning av döva och hörselskadade elever, vad skulle du då välja att delta i?

Marcar apenas uma oval.

- ( ) Kort kurs (e-baserat lärande)
- ( ) Seminarium/workshop
- ( ) Utbyta kunskaper och diskutera med andra lärare
- ( ) annat

Tack så mycket för ditt deltagande!

Este conteúdo não foi criado nem aprovado pela Google.

Google Formulários
